# The spread of non‐native species

**DOI:** 10.1002/brv.70121

**Published:** 2025-12-23

**Authors:** Phillip J. Haubrock, Ali Serhan Tarkan, Irene Martín‐Forés, Stelios Katsanevakis, Ronaldo Sousa, Ismael Soto, Andy J. Green, Antonín Kouba, Teun Everts, Victoria Dominguez Almela, Nadège Belouard, Cang Hui, Jamie Bojko, Victor Deklerck, Margaux Boeraeve, Franz Essl, J. Robert Britton

**Affiliations:** ^1^ Department of Life and Environmental Sciences Bournemouth University Poole, Dorset, Talbot Campus, Fern Barrow, Poole Dorset BH12 5BB UK; ^2^ University of South Bohemia in České Budějovice, Faculty of Fisheries and Protection of Waters, South Bohemian Research Centre of Aquaculture and Biodiversity of Hydrocenoses Zátiší 728/II, 389 25 Vodňany Czech Republic; ^3^ University of Lodz, Faculty of Biology and Environmental Protection, Department of Ecology and Vertebrate Zoology Banacha 12/16 90‐237 Łódź Poland; ^4^ Department of Basic Sciences, Faculty of Fisheries 48170 Kötekli/Muğla Türkiye; ^5^ School of Biological Sciences, The University of Adelaide North Terrace Campus Adelaide South Australia 5005 Australia; ^6^ TERN, School of Biological Sciences, The University of Adelaide North Terrace Campus Adelaide South Australia 5005 Australia; ^7^ Department of Marine Sciences University of the Aegean University Hill 81100 Mytilene Greece; ^8^ Centre for Molecular and Environmental Biology (CBMA)/ARNET‐Aquatic Research Network & IB‐S, Institute of Science and Innovation for Bio‐Sustainability, Department of Biology University of Minho 8 Campus Gualtar 4710‐057 Braga Portugal; ^9^ Department of Conservation Biology and Global Change Estación Biológica de Doñana (EBD), CSIC Américo Vespucio 26 41092 Sevilla Spain; ^10^ Genetic Diversity, Research Institute for Nature and Forest (INBO) Gaverstraat 4 Geraardsbergen 1000 Belgium; ^11^ Biology Department KU Leuven Kasteelpark Arenberg 31 Heverlee 3001 Belgium; ^12^ School of Geography and Environmental Sciences, University of Southampton Southampton, University Road Southampton SO17 1BJ UK; ^13^ Université de Rennes, CNRS, ECOBIO [Ecosystèmes, biodiversité, évolution] UMR 6553 ECOBIO, Campus de Beaulieu, Bât. 14, CS 74205, 263 av. du Général Leclerc 35000 Rennes France; ^14^ Centre for Invasion Biology, Department of Mathematical Sciences Stellenbosch University Stellenbosch 7602 South Africa; ^15^ School of Health and Life Sciences, Teesside University Middlesbrough TS1 3BX UK; ^16^ National Horizons Centre, Teesside University Darlington DL1 1HG UK; ^17^ Meise Botanic Garden Nieuwelaan 38 1860 Meise Belgium; ^18^ Division of BioInvasions, Global Change & Macroecology University Vienna Rennweg 14, 1st floor 1030 Vienna Austria

**Keywords:** Allee effects, biological invasions, diffusion, dispersal, invasion front, invasiveness, non‐native species

## Abstract

The global redistribution of species through human agency is one of the defining ecological signatures of the Anthropocene, with biological invasions reshaping biodiversity patterns, ecosystem processes and services, and species interactions globally. Here, we review the facets underlying the spread of non‐native species – the key process by which introductions translate into large‐scale invasions. In particular, we synthesise the ecological, evolutionary, and anthropogenic mechanisms underpinning the spread of non‐native species, highlighting how dispersal, recruitment, and establishment interact across spatial and temporal scales. We examine the dynamics of non‐native species spread in animals, plants, fungi, and pathogens, as well as across terrestrial, freshwater, and marine realms, with particular attention to the dynamics and processes modulating spread. We further evaluate essential phenomena of non‐native species spread, such as the role of invasion fronts, Allee effects, propagule pressure, interactions with environmental change, landscape properties, and biotic interactions. We then outline how spread can be measured, modelled, and predicted using tools ranging from classical diffusion models to cutting‐edge Artificial Intelligence and individual‐based simulations. By offering a cross‐system and cross‐scale synthesis, this review advances the theoretical and practical understanding of non‐native species spread for supporting policy and management.

## I. INTRODUCTION

Invasion science contributes to the broader goal of examining how species introduced beyond their native ranges establish, spread and interact with novel environments, communities, and disturbance regimes, often revealing key mechanisms of ecological resilience, adaptability, and system change (Richardson & Pyšek, 2006; Simberloff *et al*., 2013). Investigating the spatio‐temporal dynamics underlying how non‐native species spread is fundamental to invasion science and is often seen as the key criterion for defining a species status as ‘invasive’ (*sensu* Pyšek *et al*., 2004; Blackburn *et al*., 2011; Soto *et al*., 2024). However, it also has critical relevance to ecology, biogeography, and evolutionary biology as it provides a better understanding of the mechanisms underlying range expansion, community restructuring, and responses to anthropogenic stressors (Wilson *et al*., 2009; Gallien *et al*., 2010). For instance, analyses of spreading non‐native species offer a unique view into the spatiotemporal dynamics of biological invasions and can inform preventive management efforts (Williamson, 1996). Classic examples include freshwater fish, such as the pumpkinseed (*Lepomis gibbosus*) or rainbow trout (*Oncorhynchus mykiss*), and marine invertebrates like European green crab (*Carcinus maenas*) or terrestrial plants, such as black wattle (*Acacia mearnsii*), which have been repeatedly introduced across continents and ecosystem types and could enable comparative insights into invasion dynamics. Indeed, the study of biological invasions provides a unique opportunity to observe ongoing ecological and evolutionary processes in real time (Cox, 2004; Sax, Stachowicz & Gaines, 2005; Sax *et al*., 2007), given some species have been introduced repeatedly in large numbers from different native populations and into multiple locations, allowing for robust comparisons across temporal and spatial scales. Biological invasions also offer insights into how spread capacity and mechanisms facilitate or limit species distributions – an increasingly relevant topic for both science and management amid current and projected environmental change (Hellmann *et al*., 2008; Moran & Alexander, 2014; Liu *et al*., 2023).

Accordingly, and especially following the publication of Elton's (1958) seminal book, ecologists have been increasingly interested in the shifting dynamics of non‐native species ranges and their boundaries, which has since emerged as a central theme in invasion science (Pyšek & Richardson, 2010; Lockwood, Hoopes & Marchetti, 2013). This interest is particularly pronounced in the context of global environmental change and its role in altering species distribution, persistence, and ecological impacts (Essl *et al*., 2019; Carneiro *et al*., 2025), including impacts caused by non‐native parasites (Bojko, Dunn & Blakeslee, 2023*b*). Although the threat posed by biological invasions is well recognised (IPBES, 2023; Roy *et al*., 2024), assessing risks and their ecological, economic and social impacts remains challenging given their fundamental link to the species' ability to spread, establish, and persist in novel environments. Moreover, the spatial spread of non‐native species – here defined as the movement of a species at any speed and any direction within or beyond its introduced range after an initial introduction, followed by progressive population establishment (Williamson, 1996) – is a complex and context‐dependent process, varying across taxa, ecosystems, and spatiotemporal scales. It is influenced by an interplay of abiotic factors, including habitat connectivity, environmental heterogeneity, and disturbance regimes. Biotic and human factors are also important, including reproductive strategies, genetic diversity, dispersal capacity (including help from animal and human vectors), behaviour, trophic interactions, and ecological plasticity, all of which can either facilitate or constrain range expansion (Olden, Poff & Bestgen, 2006; Kolar & Lodge, 2001; Guisan & Thuiller, 2005; Catford, Jansson & Nilsson, 2009; Cox, 2013; Bradley *et al*., 2024). Consequently, spatial changes in a non‐native species' range can trigger cascading ecological effects, such as hybridisation with native species, trophic disruption, biogeochemical alterations, and changes in physical habitat structure, as well as a series of economic and social impacts (Sousa, Gutiérrez & Aldridge, 2009; Strayer, 2010; Gutiérrez, Jones & Sousa, 2014; Ricciardi *et al*., 2017; Soto *et al*., 2025). However, recent studies have revealed that the spread of non‐native species can differ notably across space and time (Haubrock *et al*., 2022; Soto *et al*., 2023*a*). This realisation has led to a conceptual shift in invasion science to emphasise the need to focus on population dynamics, both spatially and temporally, as the most appropriate ecological unit for assessing invasion risk and impact (Haubrock *et al*., 2024; Sousa, Nogueira & Padilha, 2024).

Understanding how organismal interactions with physico‐chemical and biological environments shape the patterns and dynamics of life across scales is central to ecology (Levin, 1992; Loreau, Naeem & Inchausti, 2001). The spread of non‐native species, including the formation, stability, and transition of their ranges, offers key insights into ecosystem resilience amid global change (Strayer, 2010; Ricciardi *et al*., 2017). Accordingly, this review synthesises current knowledge on the conceptual foundations of non‐native species spread, the metrics used to quantify it, and the modelling approaches and technology developed to capture ongoing, past, and future spread dynamics.

## II. MECHANISMS, DRIVERS, AND DYNAMICS

### (1) Describing non‐native species spread

Traditionally, the spread of non‐native species has been compared to waves that occur when a stone is dropped on a lake (Williamson, 1996). However, a widely accepted threshold to define ‘spread’ is currently lacking (but see Richardson *et al*., 2020). Spread largely remains as a binary concept – either a species is considered to spread or not – which can hinder effective management of biological invasions and complicate the allocation of limited resources. Thus, holistically defining the spread of non‐native species and all its associated complexities requires moving beyond the conventional, linear invasion framework of transport, introduction, establishment, and spread (Blackburn *et al*., 2011). Following a primary introduction event into a novel environment, either intentionally (e.g. aquaculture, biocontrol, horticulture) or unintentionally (e.g. *via* ballast water, ornamental trade, or cargo contamination), a non‐native species may survive, reproduce, and form a self‐sustaining population locally, at which point it is considered established (*sensu* Soto *et al*., 2024). Following establishment, the next phase, often referred to as the final stage of the invasion continuum, involves the spread or spatial expansion of the population (potentially boosted by secondary introductions) within the new range, often driven by the remaining local suitable, yet uncolonised habitats or when opportunities arise (Blackburn *et al*., 2011; Haubrock *et al*., 2024). Indeed, these stages of invasions and patterns of spread of non‐native species have been compared to human infectious pathogens (Nuñez, Pauchard & Ricciardi, 2020; Vilà *et al*., 2021). Additionally, describing the spread of species is even more complex due to the inherent variability among populations of the same species. For instance, the range expansion of the house sparrow *Passer domesticus* in Australia ranged from 6 to over 100 km per year, while averaging 17 km per year in the USA and 28 km per year in Europe (van den Bosch, Hengeveld & Metz, 1992; Williamson, 1996). This confirms that context matters, and factors such as local population features, invasion time, points of introduction, or invasion vector may affect the rate of spread, even among individuals from the same population. Here, only (*i*) introductions and dispersal through human agency outside a species' native range and (*ii*) secondary dispersal (i.e. natural movement of introduced non‐native species after an initial introduction) are considered (*sensu* Soto *et al*., 2024). This means that the spread of native species and populations without human interference is not considered spread *sensu stricto* in the context of biological invasions. Thus, in its most simplistic form, non‐native species spread is typically characterised by a triphasic dynamic: an initial lag phase with limited expansion, a phase of rapid range expansion, and finally a saturation phase in which spread decelerates as suitable habitats become occupied (Hastings *et al*., 2005; Arim *et al*., 2006). While these sequences remain a useful heuristic, a detailed analysis of the inherently complex, context‐dependent, and spatially and temporally hierarchical nature of biological invasions is warranted.

**Fig. 1:**
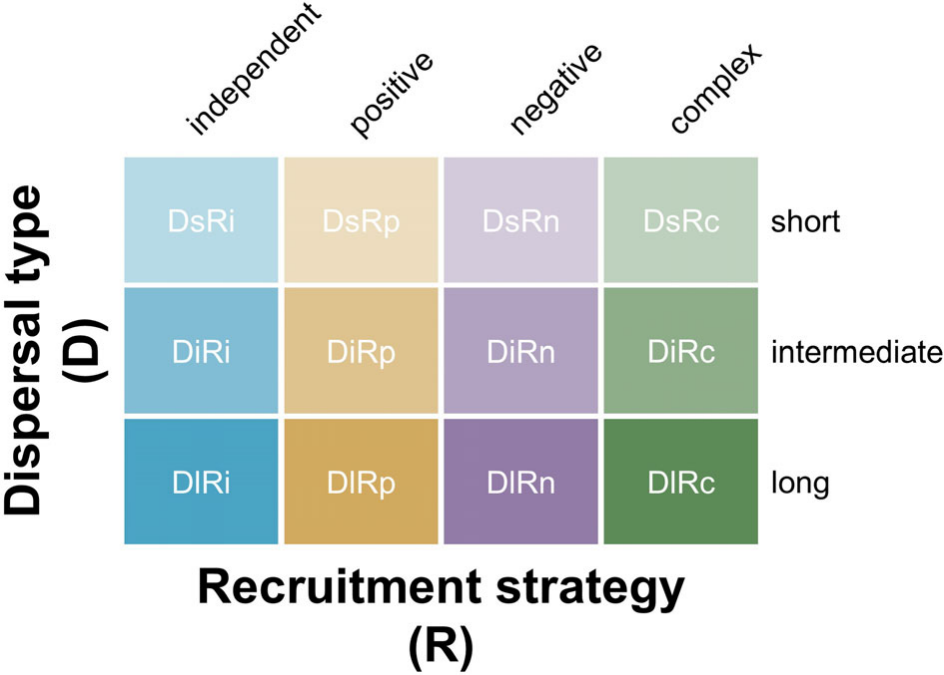
Conceptual classification of non‐native species spread as a function of dispersal type and recruitment strategy. Spread arises from the interaction between dispersal (Ds, short; Di, intermediate; Dl, long‐distance) and recruitment (Ri, density independent; Rp, positively density dependent; Rn, negatively density dependent; Rc, complex strategies). Each cell represents a distinct spread scenario, combining the spatial reach of movement with the demographic outcomes at the colonisation site.

As spread encompasses a non‐native species' range expansion from points of introduction (Hulme *et al*., 2008; Wilson *et al*., 2009), it is always secondary in nature and depends critically on the frequency and success of dispersal events, as well as the distance to the native range. This ‘dispersal effectiveness’ is defined as the process by which propagules of a non‐native species are not only transported to a new region, but also establish successfully through recruitment into adult stages and subsequent reproduction (Lawson Handley *et al*., 2011; Auffret *et al*., 2017). Although often used interchangeably, dispersal and spread are not synonymous. Dispersal refers to the movement of propagules, while spread – defined here as range expansion of a non‐native species – is the outcome of both dispersal and successful recruitment or establishment at the destination site. This distinction is crucial, as many species disperse without establishing, and spread dynamics emerge from the interaction between dispersal kernels and recruitment strategies. Indeed, while dispersal is a necessary component of non‐native species spread, it is not sufficient on its own. Successful spread also depends on recruitment, i.e. the ability of individuals to survive, grow, and reproduce, in newly reached areas (Ling *et al*., 2008; Gutowsky & Fox, 2012). Spread, therefore, emerges from the interaction between dispersal and recruitment processes across space. This relationship can be expressed formally as:

$$n\left(x,t+1\right)=\int_{y\in E}f\left(n\left(y,t\right)\right)k\left(y,x\right)\mathrm{dy}$$

where *t* is time, $k\left(y,x\right)$ is the dispersal kernel (i.e. the probability of moving propagules from location *y* to *x*), and $f\left(n\left(y,t\right)\right)$ is the recruitment function (which may be density independent or reflect positive/negative density dependence, or complex life cycles, and mostly also contextual to the environmental conditions in location *y*). This framing emphasises that dispersal alone does not constitute spread; rather, spread results when dispersal leads to successful recruitment and population growth in novel habitats (Fig. 1).

**Fig. 2:**
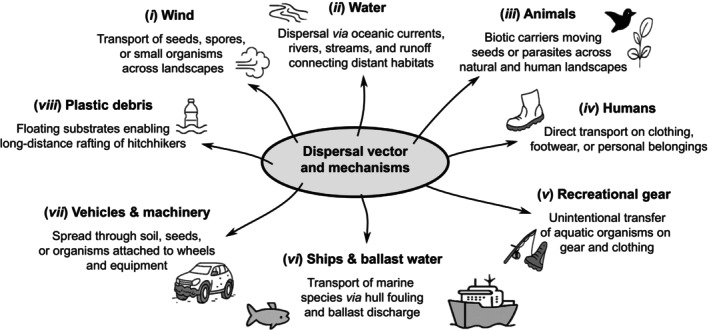
Categorisation of dispersal vectors. Examples shown are means of transport (vectors) and mechanism of propagule dispersal.

**Fig. 3:**
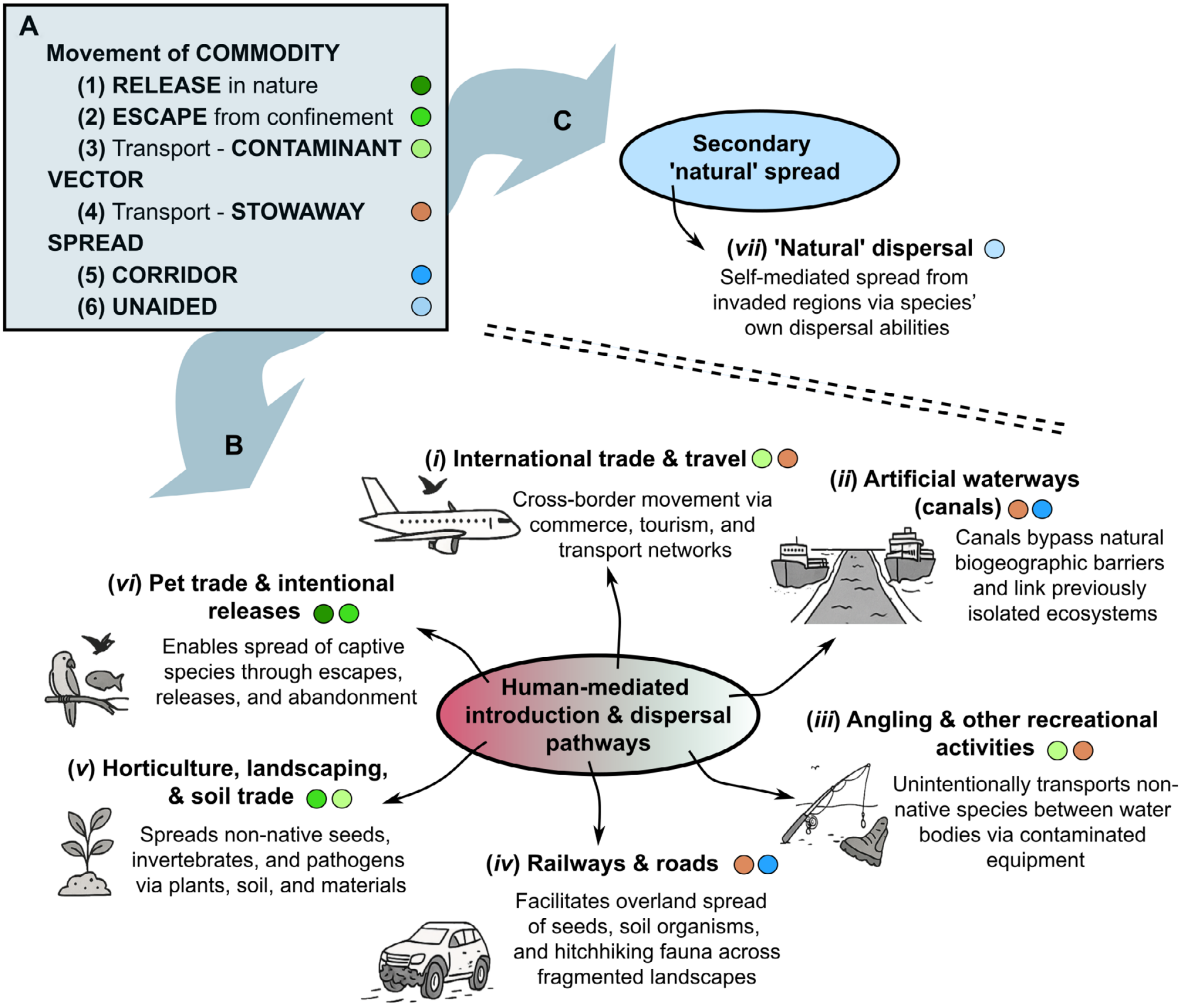
Categorisation of dispersal pathways according to the Convention of Biological Diversity (A) based on on Hulme (2009) and CBD (2014) to illustrate the distinction between key anthropogenic pathways through which non‐native species are introduced and dispersed (B) and how non‐native species spread naturally (C) beyond their native ranges. Example human‐mediated pathways shown include international trade and travel (*i*), artificial waterways (*ii*), angling and recreational equipment (*iii*), railroads and roads (*iv*), horticulture, landscaping, and soil trade (*v*), and the pet trade (*vi*, including intentional releases).

Importantly, even non‐native species that fail to establish at the original introduction site may still disperse, either actively or passively, and establish elsewhere (Brown & Barney, 2021). To this end, non‐native species may spread (or may be spread) through a range of mechanisms that broadly fall into two categories: natural dispersal and human‐mediated movement (Catford *et al*., 2009; Faulkner, Hulme & Wilson, 2024). Natural dispersal can encompass short‐distance diffusion, intermediate dispersal, and long‐distance dispersal (Table 1). Short‐distance dispersal can occur through the leading edge or gradual range expansion by a species' own means, including active movement (e.g. swimming, walking, flying) or through the use of passive vectors (e.g. wind, water currents, or species‐mediated transport; Mason, Baruzzi & Lashley, 2022). Intermediate dispersal can occur naturally *via* animal vectors during their daily movements by endozoochory [i.e. dispersal *via* ingestion by animals, including fish eggs inside waterfowl (Guy‐Haim *et al*., 2017; Lovas‐Kiss *et al*., 2020, 2023)] or epizoochory [i.e. dispersal *via* attachment to the outside of animals such as plants or snails attached to amphibians or waterfowl (Saito *et al*., 2023; Gould & Valdez, 2024)] (Fig. 2). However, even occasional long‐distance dispersal of established non‐native species (e.g. transoceanic movements) can be natural, i.e. of seeds or eggs attached to birds during migrations or after natural disasters (e.g. tsunamis; Carlton *et al*., 2017). Similarly, human‐mediated dispersal can also occur across short, intermediate, and long distances (Gippet *et al*., 2019). For example, local dispersal may result through diverse pathways (e.g. from recreational activities), whereas intermediate and long‐distance dispersal often involve the transport of goods, ballast water, or infested materials across regions or continents (Fig. 3). Non‐native insects may expand *via* active dispersal (e.g. flight) or passive mechanisms (e.g. hitchhiking or naturally on winds) (Kulessa *et al*., 2024) but can also appear in distant regions through anthropogenic means, such as the transport of infested plant materials (Liebhold *et al*., 2012). On some occasions, rare natural processes such as river flooding can also drive long‐distance dispersal (Everts *et al*., 2025). Recognising that local, intermediate, and long‐distance dispersal can simultaneously contribute to overall spread (i.e. stratified dispersal) is critical for accurately interpreting and predicting invasion dynamics (Lockwood, Cassey & Blackburn, 2005; Rius & Darling, 2014; Sherpa *et al*., 2020). Notably, however, the dispersal of individuals does not necessarily contribute to range expansions (Sepulveda, 2018). This is because, for instance, vectored dispersal (natural or anthropogenic) may lead individuals or propagules through or to unsuitable environments (e.g. extreme temperatures on aircraft surfaces, limited access to food in shipping containers) where they cannot survive (Renault *et al*., 2018). Species dispersal is often structured by source–sink dynamics, arising from site‐level imbalances in immigration and emigration ratios (Hudgins *et al*., 2023). In this framework, source populations actively contribute to the spread of a species by producing excess individuals that disperse outward, while sink populations are maintained primarily through immigration and are unable to sustain themselves without a continued influx (Dauphinais *et al*., 2018; Belouard *et al*., 2019; Peniston *et al*., 2024).

**Table 1:** Types of dispersal contributing to non‐native species spread, with associated mechanisms, examples, scales, and causes.

Dispersal type	Mechanism	Example	Spatiotemporal scale	Typical cause	Corresponding category by Faulkner *et al*. (2024)
Short‐distance dispersal	Continuous colonisation, i.e. diffusion from an initial point of introduction	Short flights, crawling, or short‐range wind dispersal	Daily to seasonal; metres to a few kilometres	Autonomous movement of individuals or vectors	7: Moved by a human‐introduced biotic vector; 8: Moved by a natural abiotic vector; 9: Moved by a natural biotic vector; 10: Self‐propelled
Intermediate dispersal	Often biotically assisted (e.g. daily movements by birds, mammals) or weather related (e.g. storm events) or human mediated (e.g. recreational boats)	Bird‐mediated insect dispersal, rafting, extreme wind events, floods	Episodic, tens to a couple of hundreds of kilometres	Natural but less frequent events than local diffusion	3*: Moved accidentally with a commodity; 4*: Moved accidentally during transport; 5*: Moved along a human‐built corridor; 7: Moved by a human‐introduced biotic vector; 8: Moved by a natural abiotic vector; 9: Moved by a natural biotic vector
Long‐distance dispersal	Includes both natural and human‐mediated vectors (e.g. bird migrations, or vagrancy)	Windstorms, tsunamis, oceanic drift, plastic rafting, or global trade	Rare but consequential; a few hundreds to thousands of kilometres	Natural rare events or anthropogenic transport	1: Moved intentionally for release; 2: Moved intentionally for use in captivity or cultivation; 3*: Moved accidentally with a commodity; 4*: Moved accidentally during transport; 5*: Moved along a human‐built corridor; 6: Moved by a human‐made abiotic vector; 7: Moved by a human‐introduced biotic vector; 8: Moved by a natural abiotic vector; 9: Moved by a natural biotic vector

* Categories 3, 4, and 5 can apply to short‐distance dispersal, but their practical relevance is limited, as such events are less frequent, less documented, and typically less consequential for spread, thus commonly describing intermediate to long‐distance, often global, movements.

However, if spread is defined more broadly as the range expansion of a non‐native species beyond the initial point of introduction within a novel area, then secondary human‐mediated introductions, outgoing from an earlier introduction site but also through further introductions from the species' native ranges (e.g. Bertelsmeier & Ollier, 2021), must also be considered as range expansion (Richardson *et al*., 2000; Horak *et al*., 2013; Pyšek & Richardson, 2010; Soto *et al*., 2023*b*; Tarkan *et al*., 2024*b*). Acknowledging that both processes occur is crucial, as human‐mediated secondary introductions from the native range (as part of propagule pressure; Lockwood *et al*., 2005) allow non‐native species to bypass human, geographic, or ecological barriers or established range boundaries, resulting in abrupt establishment events in direct proximity or possibly far from their previously invaded ranges (Essl *et al*., 2015; Hulme, 2009; Oficialdegui *et al*., 2019). In particular, secondary dispersal events complicate the distinction between initial introduction sites and places of establishment and may lead to multiple expanding invasion foci within an invaded range (Lockwood *et al*., 2005; Lejeusne *et al*., 2014; Shigesada & Kawasaki, 2016; Tedeschi *et al*., 2025). Moreover, repeated introductions, particularly when coming from different source populations, may lead to admixture, which in turn may affect and often enhance the invasion potential of founder populations (Dlugosch *et al*., 2016).

Even when spread is initially gradual and rapid, it may transition into pulsed or clustered expansions if dispersal becomes maladaptive or energetically costly in certain regions (Travis *et al*., 2009; Urban *et al*., 2008) or when expanding populations from independent introduction sites merge (Wilson *et al*., 2009). The spread of non‐native species often unfolds in a patchy, irregular manner, shaped by stochastic events, environmental heterogeneity, dispersal barriers, eco‐evolutionary dynamics, and fluctuations in dispersal scales (Lewis, 1997). These localised ‘patches’ of non‐native populations within an invaded range can also retract and disappear (Simberloff & Gibbons, 2004), or they may gradually expand, merge, or be connected *via* rare long‐distance dispersal events (Shigesada & Kawasaki, 2016). Such events can result in the establishment of new populations far ahead of the main invasion front, leading to rapid or even accelerating spread. This phenomenon is commonly described as stratified diffusion (Shigesada, Kawasaki & Takeda, 1995) and results from mixed dispersal distances rather than evolutionary processes (such as spatial sorting). It has been observed in a wide array of non‐native species, including plants, invertebrates, and vertebrates (Kamata, 2006). At the core of these dynamics lie dispersal mechanisms and species–environment interactions, which are key determinants of invasion speed and spatial patterning (Nathan *et al*., 2008).

During ongoing range expansions of non‐native species, dispersing individuals often encounter unoccupied habitats at the range edge, where selection pressures differ markedly from those in core populations (Travis & Dytham, 2002; Burton, Phillips & Travis, 2010). Traits favouring rapid dispersal often evolve rapidly at invasion fronts, despite potential fitness costs (Brown *et al*., 2007; Peischl, Kirkpatrick & Excoffier, 2015), through spatial sorting mechanisms distinct from natural selection (Shine, Brown & Phillips, 2011), especially in patchy habitats (Williams, Kendall & Levine, 2016), while in core populations competitiveness may be favoured (Messager & Olden, 2019). This can accelerate range expansion beyond environmental limits (Ochocki & Miller, 2017). The spread of non‐native species is thus a complex and multifaceted process that requires insights from mathematics, physiology, physics, genetics, movement and spatial ecology, community ecology (i.e. demography and population ecology), and invasion science. Thus, it is a process inherently difficult to describe in a singular, universal, or simple way, as its interpretation depends on scale, context, and the underlying mechanisms driving movement and range expansion.

#### (a) How non‐native animals spread

Many animal species disperse through a combination of pathways and inherent dispersal modes, such as active movement and passive transport (Everts *et al*., 2025). Natural dispersal in animals can be active, such as through flying, swimming, or walking to new areas, or passive, such as hitchhiking (i.e. phoretic dispersal) or drifting on currents, rafts or winds. Dispersal capacity is highly species or population specific, reflecting variation in mobility, life history, and reliance on external vectors (Clobert, Ims & Rousset, 2004). Among vertebrates, terrestrial mammals and reptiles often disperse through active locomotion, typically over short to moderate distances, but occasionally also using rafts, *via* hitchhiking, or trade as exotic pets (Kraus, 2007). Conversely, birds can cover large distances rapidly, sometimes crossing continents during migration or post‐release expansion (Blackburn, Lockwood & Cassey, 2009). Invertebrate dispersal strongly relies on the combination of both active and passive dispersal strategies (Sherpa *et al*., 2020). Many insects can fly and self‐disperse efficiently, while other invertebrates, like molluscs or crustaceans, are more reliant on water flow, currents, vectors, or human transport [e.g. *via* ballast water, hull fouling, aquaculture, or landscaping material (Pergl *et al*., 2017; Urban & Leach, 2023; Oficialdegui *et al*., 2025)]. Non‐native aquatic organisms, particularly fishes, exemplify this interplay as they are often introduced in large numbers and can disperse *via* multiple mechanisms, from active swimming and natural connectivity to human‐mediated pathways, such as stocking, aquaculture escapes, or transport in ballast water, making these mechanisms especially consequential for invasion dynamics (Bernery *et al*., 2024). Additionally, dispersal of some invertebrates, such as certain ants, aphids, and spiders, is assisted by wind, which can lead to particularly rapid rates of spread (Ollier & Bertelsmeier, 2024). Animal species expressing range expansion can thus use a combination of natural and human‐mediated mechanisms.

The spread of non‐native animals is often faster than plants, where the most mobile individuals lead the front (i.e. ‘spatial sorting’) and confer an accelerating spread rate to the population (Phillips *et al*., 2008, 2008). The distance a species can spread thus becomes a function of several factors. These include the species' available modes of dispersal (Ptatscheck & Traunspurger, 2020), its size (Jenkins *et al*., 2007), behaviour (Weis & Sol, 2016), the time of invasion, and the invaded environment (Williamson, 1996). For example, while the area in e.g. ponds or lakes is limited, the dispersal of a non‐native freshwater species can occur in all directions – radiating outward along the shoreline and across open water – whereas in riverine ecosystems, spread is constrained to a linear path (unless mediated by canals; Goldberg *et al*., 2010). In marine environments, however, many benthic animals are sessile or have limited mobility during most of their life cycle (Pechenik, 2015). Consequently, both their dispersal and spread depends largely on larval transport *via* ocean currents, often supplemented by human‐mediated movement through ballast water or hull‐fouling (Bailey, 2015).

Aside from interspecific differences, a distinctive feature of how animals spread lies in intraspecific variation in dispersal mechanisms, rates, and frequencies. Animal species can exhibit sex‐biased dispersal, where males and females differ in the timing, extent, and mode of movement (reviewed in Li & Kokko, 2019). In most mammals, males typically disperse more frequently and over greater distances than females, whereas in birds, the reverse is often true (Greenwood, 1980; Fandos *et al*., 2023) and in fishes and reptiles both male‐ and female‐biased dispersal patterns occur (Hutchings & Gerber, 2002; Taylor *et al*., 2003; Keogh, Webb & Shine, 2007; Olsson & Shine, 2003). Notably, wing polymorphism with a dichotomous sex difference in insects can give rise to an entirely wingless sex, severely limiting their dispersal potential (Wahlberg *et al*., 2010). In species with territorial behaviour, one sex may prioritise locating and defending a territory, while the other follows to establish residency or mate (Trochet *et al*., 2016). Dispersal may also vary across life stages. In some amphibian species, cannibalistic behaviour of adults can incentivise juveniles to disperse rapidly upon completing metamorphosis (DeVore *et al*., 2021). Additional intraspecific behavioural and physiological traits can also influence dispersal (Myles‐Gonzalez *et al*., 2015). This so‐called ‘behavioural invasion syndrome’ describes sets of inter‐individual differences in behaviour that consistently occur together (Sih, Bell & Johnson, 2004; Galli *et al*., 2023). This includes individuals that exhibit higher exploratory behaviour and risk‐taking being more likely to disperse further and colonise new areas, potentially gaining access to better resources or mates (Burstal *et al*., 2020; Galib *et al*., 2022).

#### (b) How non‐native plants spread

Many plant species can disperse by multiple mechanisms, a phenomenon known as polychory [e.g. combining wind with external and internal transport by vertebrates, including humans (Green, Baltzinger & Lovas‐Kiss, 2022; González‐Varo *et al*., 2024)]. Indeed, being sessile, plants spread by dispersing their propagules, i.e. seeds, spores, or vegetative parts. Some species rely on their own mechanical means (autochory) for either passive dispersal through gravity fall (barochory) or explosive dispersal by ejecting seeds (ballochory, e.g. *Impatiens* spp.; van Rheede van Oudtshoorn & van Rooyen, 1999). However, most plants rely heavily on external vectors, such as wind (anemochory) (van Rheede van Oudtshoorn & van Rooyen, 1999), water currents (hydrochory) (Nilsson, Gardfjell & Grelsson, 1991; Nilsson *et al*., 2010) or animals (epi‐ and endozoochory) (Iluz, 2010), but most notoriously as the consequence of anthropogenic activity (anthropochory) (Hodkinson & Thompson, 1997). This broad scope of dispersal mechanisms is not only applied to vascular plants but also to other members of the Plantae and Chromista kingdoms. For instance, ferns primarily disperse *via* spores, which are often carried by wind or water. Similarly, many cryptogams (e.g. mosses) rely on spores for reproduction and dispersal. Algae have a wide range of dispersal mechanisms, including water currents, attachment to animals, human‐mediated through fishing gear or as contaminants in transport, or aquarium products, allowing them to colonise new aquatic habitats. Spread of marine algae often involves dislodged adult thalli or fragments rather than planktonic propagules, which typically have limited dispersal potential (Santelices, 1990). Many non‐native macroalgae can float or attach to debris, facilitating long‐distance drift, and in species that can reproduce vegetatively or parthenogenetically, single fragments can establish new populations far from their source, e.g. *Sargassum muticum* appears to have spread 1,100 km along the entire coast of California in a single step (Deysher & Norton, 1981).

Unintentional human‐mediated transport of plants includes, among others, the movement of contaminated soil and machinery carrying seeds or propagules as stowaways, whereas deliberate introduction is mostly associated with the intentional introduction of crops, horticulture, and ornamental plants (Hulme *et al*., 2008; Montagnani *et al*., 2022). The spread of non‐native plants can therefore be decoupled from natural dispersal syndromes (i.e. sets of plant traits associated with specific dispersal mechanisms), often displaying much larger range expansion when associated with anthropogenic activities than would be expected by natural dispersal (Moyano *et al*., 2022). Effectiveness of plant spread is further modulated by intrinsic factors, such as seed morphology, release height, phenology, and propagule pressure, with spread rate often being associated with the production of numerous small persistent seeds and the capacity for vegetative regeneration, including spread *via* rhizome fragments (Martínez‐Ghersa & Ghersa, 2006; Mason *et al*., 2008; Klinerová, Tasevová & Dostál, 2018). In addition, clonal growth can facilitate centrifugal horizontal spread, bypassing seedling establishment filters (Herben & Klimešová, 2020). Importantly, plants often rely on multiple dispersal pathways due to their innate immobility. These so‐called ‘bet‐hedging’ strategies are generally associated with the production of dimorphic fruits (Childs, Metcalf & Rees, 2010). Examples of this are *Leontodon saxatilis* and *Hypochaeris glabra* (Martín‐Forés *et al*., 2018*a*), two annual plant species that are widely distributed in the Mediterranean biome. *Leontodon saxatilis* produces two types of fruits (i.e. achenes), the external ones are smooth or finely muricate and not beaked, exhibit higher germination rates, and are mainly expected to grow in the vicinity of the mother plant. By contrast, the inner achenes are muricate and have a beak with a pappus for undergoing wind dispersal (Martín‐Forés *et al*., 2017). A single non‐native plant species might have a local diffusion through short‐distance seed rain and occasional long‐distance dispersal events that leapfrog it into more distant, uncolonised regions (Wichmann *et al*., 2009). In addition, intraspecific trait variation often modulates a plant's seed output and its ability to spread. For example, dioecious species, such as *Juniperus thurifera*, display major differences regarding dispersal mechanisms between male and female trees, leading to sex‐dependent structure within populations. Specifically, male trees produce pollen that can be wind dispersed over long distances, while female trees produce fleshy fruits or nuts that attract frugivorous fauna, leading to seed dispersal primarily in the vicinity of their canopies (Martín‐Forés *et al*., 2022). Individuals in a given population can present marked differences with regards to biomass, with consequences for reproductive output, dispersal potential, and growth dynamics (Martín‐Forés *et al*., 2018*b*), all of which ultimately affect dispersal capacity. For some pines (e.g. *Pinus sylvestris* and *P. radicata*), seed mass, wing area, and seed terminal velocity are determinants of their dispersal capacity, even influencing subsequent emergence rate and seedling growth in some instances (Debain, Curt & Lepart, 2003; Wyse, Hulme & Holland, 2019). Clonal species, such as *Phragmites australis*, can also show intraspecific variation in multiple traits simultaneously, affecting their capacity for vegetative growth and spread (Ren *et al*., 2020).

Humans are extremely effective vectors for plant propagules, facilitating the invasion of many plants by agriculture, horticulture, construction works, fisheries, and ornamental purposes. Moreover, unintentional dispersal by humans has enabled non‐native plants to reach even the most remote areas of the planet (Chown *et al*., 2012; Ware *et al*., 2012; Liedtke *et al*., 2020). These events contribute to leptokurtic dispersal distributions (i.e. most spread distances are relatively short, while a few are large) and ultimately direct spread rates in plant invasions (Lewis, Petrovskii & Potts, 2016). Overland plant dispersal is further shaped by landscape features, with topography, geomorphology, prevailing winds, hydrology, and disturbance regimes influencing passive directed dispersal of plants by animals (Mason *et al*., 2022), and its directionality and scale. Climate suitability, habitat and soil properties affect post‐dispersal survival, while biotic resistance modulates establishment following introduction. Ultimately, the spread dynamics of plants, just like that of animals, emerge from the interaction between dispersal mechanism, landscape structure and permeability, and context‐dependent abiotic and biotic filtering (Robledo‐Arnuncio *et al*., 2014).

#### (c) How non‐native fungi spread

Many fungal species can disperse through a combination of active movement (hyphal expansion) and transport of propagules (including sexual or asexual spores, hyphal fragments, sclerotia) by biotic or abiotic vectors. While hyphal expansion mostly happens at small spatial scales (cm–m scale), the existence of genets of hundreds or even thousands of m^2^ (Bonello, Bruns & Gardes, 1998; Bendel, Kienast & Rigling, 2006) suggests that some species are capable of spreading in this way given sufficient time. Biotic vectors of fungal propagules include humans, arthropods, earthworms, mammals, and birds, while the most important abiotic vectors are wind and water. Apart from a few deliberate introductions for mycorrhization or biological control, introductions of non‐native fungi predominantly happen unintentionally (Desprez‐Loustau, 2009; Monteiro *et al*., 2020, 2022). How non‐native fungi subsequently spread depends on their lifestyle and dispersal mechanisms.

Mutualists like mycorrhizal fungi and pathogens depend on the presence of a suitable host. The large majority of non‐native ectomycorrhizal fungi have been co‐introduced with their host species, often tree species of commercial value, such as *Pinus* spp. or *Eucalyptus* spp. (Vellinga, Wolfe & Pringle, 2009). However, most of them seem unable to spread to new regions because they only associate with their introduced host (Vellinga *et al*., 2009) or because they depend on a non‐native mammal to disperse their spores (Wood *et al*., 2015). For example, in regions where *Pinus* or *Eucalyptus* have been introduced, non‐native mammals, such as squirrels, deer, or wild boar can act as spore dispersers, facilitating the spread of their associated fungi (Policelli *et al*., 2022). Whether an introduced ectomycorrhizal fungus is able to spread to local hosts thus depends on its host specificity and dispersal strategy and on the local plant community, presence of propagule vectors, and the resistance to invasion of the native ectomycorrhizal fungal community (Vellinga *et al*., 2009). For more on the spread of pathogenic fungi (and other pathogenic organisms), see Section II.1.*d*.

On the other hand, the spread of non‐native saprotrophic fungi depends on the presence of a suitable substrate, their dispersal strategy, and local abiotic conditions. One notable group of non‐native saprotrophic fungi are found on wood chips and mulch in various parts of the world (e.g. *Agrocybe putaminum*, *Clathrus archeri*, *Leratiomyces ceres*) (Shaw & Kibby, 2001; Vellinga, 2008). The origin of these species is largely unknown and while their introduction in various parts of the world is most likely due to human vectors, their local spread probably happens both through human vectors (e.g. transport of wood chips and mulch) and natural spore dispersal by wind or insects. Most non‐native saprotrophic fungi are predominantly found in disturbed habitats (buildings, gardens, parks, etc.) and show limited spread into natural areas (Vizzini, Zotti & Mello, 2009; Kauserud *et al*., 2012; Fraiture & Di Giangregorio, 2013). However, due to a general lack of scientific knowledge on fungal ecology and biogeography, fungi are underrepresented in invasion biology and non‐native fungi are vastly understudied (Desprez‐Loustau *et al*., 2007). The limited research that has been done on non‐native fungi is heavily biased towards pathogenic fungi, while the spread and potential ecological impact of non‐native saprotrophic or mycorrhizal fungi is generally unknown.

#### (d) How non‐native parasites spread

Biological invasions inherently include the movement of symbiotic organisms from one location to another, providing the chance for the symbiont to become invasive alongside its host (Bojko *et al*., 2023*b*). Symbionts include mutualists, commensals, and parasitic or pathogenic species that may facultatively or obligately associate themselves with a host. Symbionts can mediate the spread of their host in an array of ways (explored in Section II.4); however, the spread of a ‘non‐native parasite’ (i.e. a pathogen or parasite that is not native in a particular location or ecosystem) has its own intricacies regarding dispersal and survival, which can be understood largely through its transmission dynamics, host range, replication and evolutionary rate, and specific tolerances. Here, however, we use the term ‘non‐native parasite’ to refer to any parasitic or pathogenic organism introduced to a new environment beyond its native range, irrespective of criteria that may define its invasiveness (*sensu* Soto *et al*., 2024), as this review focuses specifically on spread. For these non‐native parasites, a multitude of factors can underpin their capacity to spread and establish in a new location or ecosystem, including: native/non‐native biodiversity (Roche *et al*., 2012); native/non‐native host density (Angulo *et al*., 2025); native/non‐native host susceptibility (Gervasi *et al*., 2017; Thines *et al*., 2023); local (micro‐)climate and climate variability (Lafferty, 2009); and competition with other parasites (Mideo, 2009). Naturally, these dynamics also mediate transmission potential, depending on the mode of transmission a non‐native parasite might use, such as horizontal (direct transmission from one organism to another), vertical (germ‐line infection), or perhaps multi‐host (transmission through multiple hosts to achieve reproductive capability, possibly *via* trophic interactions) methods. There is a broad taxonomic diversity that may fit into the non‐native parasite bracket, including viruses, bacteria, single‐celled eukaryotes, and multicellular eukaryotic organisms.

**Fig. 4:**
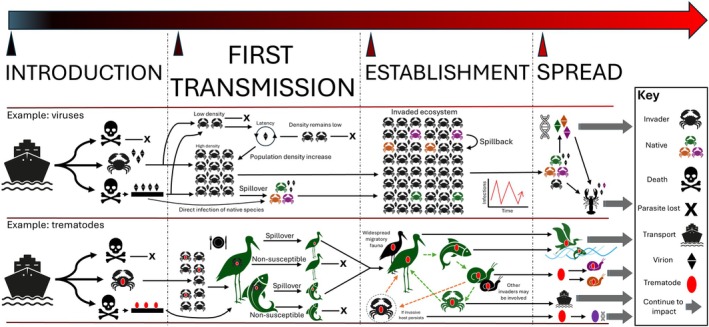
A conceptual roadmap exploring two examples based on different generalised parasite groups, a virus and a trematode, which use different transmission methods and require different circumstances upon invasion into a new area for persistence. The figure explores introduction, first transmission step, establishment within the new region, and finally spread, where the non‐native parasite may then spread to new hosts, locations, and/or undergo adaptation and evolutionary change to utilise available resources better and overcome physiological limitations. The key on the right shows the meaning of the icons. Viruses: this example includes a horizontally transmitted virus, which requires its host to survive, but can contaminate surfaces. The virus requires high density to transmit and, in the example, spills over to native species, which can then house the virus and cause it to spill back into non‐native populations.

Irrespective of its ability to cause harm (i.e. negative impacts), the capacity for a non‐native parasite to spread depends firstly on its introduction, where the host (or parasite) must survive long enough for the parasitic organism to enter the new environment (Fig. 4). If the host dies during transportation, the parasite may not be viable upon entry to a new location, unless it has the capacity to remain latent in the dead tissue or contaminate the vector surface that is driving the introductory process (Dunn *et al*., 2012). This stage of non‐native parasite introduction can include governing factors such as parasite density, parasite hardiness, and pathology. For example, a virus that produces large numbers of virions increases its chances of entering and establishing in a new region. However, if it kills its host too quickly or the virions degrade before reaching a suitable environment, the invasion may fail before it begins. Once established, the virus may spread geographically by infecting new susceptible species, mutating to broaden its host range (Lymbery *et al*., 2014). Similarly, a trophically transmitted trematode that requires multiple hosts, such as a bird, fish, snail, and crustacean, can only spread if each host interaction is ecologically viable in the invaded range (Benesh, Chubb & Parker, 2014). This means the non‐native crustacean must be both palatable to predators and physiologically compatible with the trematode. If the parasite can persist through interactions with native or co‐invasive fauna and use migratory birds or fish for dispersal, it may expand its range further, potentially adapting to new hosts and environments (Verneau *et al*., 2011). Species that do not rapidly kill their hosts – such as trematodes that encyst in the gut – are especially likely to be successfully introduced and spread. Enemy release often explores these factors surrounding the likelihood of introduction, where transportation sits as the first main barrier to non‐native parasite introduction (Miura & Torchin, 2023).

If a co‐introduced non‐native parasite survives the introduction process, a next step is to undergo its ‘first transmission’ and infect new host(s). This can depend on the prevalence of the parasite in the non‐native host, and the amount of time these infected individuals may survive to transmit the non‐native parasite to conspecifics or native fauna. If the number of propagules is large enough to overcome the density‐dependent thresholds required for the non‐native parasite to persist in the non‐native host population, initial transmission is likely to occur directly within the non‐native host population. Further, the parasite may spill over into native hosts and reach the density threshold for persistence (Dunn & Hatcher, 2015). Other possibilities include becoming latent (Dunn & Smith, 2001) or remaining inactive in the environment until the opportunity to re‐infect a population arises, such as when density thresholds increase to a size that allows viable transmission (Churcher, Ferguson & Basáñez, 2005). For example, microsporidian parasites in aquatic and terrestrial environments have infectious spore stages that can remain latent in the host or environment for many years (Dunn & Smith, 2001). This group has adapted an array of horizontal, vertical, or hybrid horizontal – vertical transmission processes to increase persistence (Bojko *et al*., 2022). If density‐dependent thresholds are not reached, or viable hosts are not present (such as the array of fauna needed for a trophically transmitted parasite such as a trematode; Fried, 2024), the non‐native parasite may become extinct at the new location. However, if the non‐native parasite is able to transmit and complete its life cycle in native or non‐native hosts, it will progress to ‘establishment’ and then begin to spread (Fig. 4).

The array of possibilities described above can lead to non‐native parasite spread, culminating in a parasite that persists in a non‐native, native, or mixed host population within an invaded ecosystem (Al‐Shorbaji *et al*., 2016, 2017). If the parasite is harmful, it would proceed to cause physiological or other impacts upon any native hosts it infects, possibly leading to mortality or behavioural change, affecting native population sizes and possibly ecosystem services. An example is the spread of crayfish plague, which causes rapid mortality in white clawed crayfish (*Austropotamobius pallipes*) and results in an immediate loss of their ecological influence (Jussila *et al*., 2015). If the non‐native parasite infects only the non‐native host, it may have a controlling effect on the population, limiting its invasiveness, among other potential consequences (Prenter *et al*., 2004). The concept of spread for a successful non‐native parasite includes its persistence in the new range, either by utilising the non‐native host or native biodiversity as a part of its transmission and is centred around its survival and range extension. It is common to see viruses, bacteria, and other parasitic groups acquire mutations that may better suit their survival in a new ecosystem. For example, COVID‐19 (SARS‐CoV‐2) has been likened to a non‐native parasite (Nuñez *et al*., 2020) and as this virus has continued to spread globally, novel mutations have occurred, resulting in new strains of the virus that are less virulent and better at transmitting and spreading (Kun *et al*., 2023).

### (2) The invasion front

**Fig. 5:**
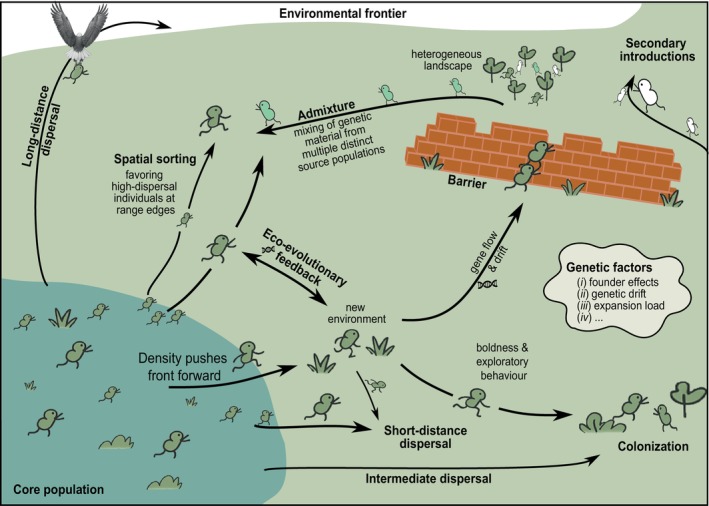
Conceptual illustration of ecological and evolutionary dynamics occurring at the invasion front, highlighting dispersal, colonisation, and trait changes during range expansions.

The invasion front represents the leading edge of a non‐native species' expanding range, namely a spatially dynamic zone where novel environments are encountered and colonised (Fig. 5). The progression of the invasion front is characterised by a continuous revisiting of the introduction and establishment processes (Blackburn *et al*., 2011), and its magnitude and direction are therefore contingent on factors influencing these processes. While often conceptualised as a continuous wave of expansion, invasion fronts may also be diffuse or fragmented, shaped by long‐distance dispersal events, dispersal corridors and barriers, or multiple, independent introductions (Mineur *et al*., 2010; Azimzade, 2022; Everts *et al*., 2025). This distinction has important implications for both ecological theory and management. A diffuse invasion front arising from repeated introductions may render localised containment strategies (e.g. firebreaks or quarantines) ineffective if the sources lie outside the scope of the current surveillance and management efforts (Zhao *et al*., 2019). Mechanistically, invasion fronts are often modelled as travelling waves, described by reaction–diffusion equations that integrate growth, dispersal, and environmental heterogeneity (Méndez *et al*., 2011). However, real‐world invasions rarely unfold in a smooth, predictable fashion. Instead, they often proceed through pulses, jumps, accelerations and decelerations, or fits and starts. Such dynamics may be driven by environmental heterogeneity (Urban *et al*., 2008) or interactions between low‐density edge populations, subject to Allee effects and stochasticity, and denser populations behind the front that ‘push’ the range forward, especially when internal processes like over‐compensatory growth or density‐dependent dispersal are in play (Sullivan *et al*., 2017). These interactions can result in unstable or fluctuating spread speeds, complicating range expansion predictions and management (Travis & Dytham, 2002).

In addition to spatial complexity, invasion fronts are also hotspots of behavioural and life‐history divergence. In animals, individuals at the front tend to be bolder, more exploratory, and show higher directional persistence, with reduced site fidelity and a greater readiness to exploit unfamiliar environments (Groen *et al*., 2012; Pizzatto *et al*., 2017*a*; Myles‐Gonzalez *et al*., 2015). While such traits may incur metabolic costs, they facilitate colonisation success. Over time, dispersal‐enhancing traits can become more common at the front *via* spatial sorting and localised selection, leading to accelerated spread (Alford *et al*., 2009). For instance, Tarkan *et al*. (2021) and Grabowska *et al*. (2021) showed that populations of two non‐native freshwater fish species at the invasion front in different streams exhibited significantly higher reproductive investment and faster growth rates compared to core populations. This suggests that phenotypic plasticity is driven by reduced competition and novel selective regimes. Thus, these findings highlight the importance of front‐specific trait expression in shaping invasion trajectories, even within limited spatial scales. Crucially, these differences are not just passive consequences of environmental conditions. They are actively shaped by eco‐evolutionary feedback, where adaptation in one patch alters selection processes of other parts of the front (Andrade‐Restrepo, Champagnat & Ferrière, 2019*a*). In addition, competitive interactions and spatial variability in habitat quality can increase the geometric ‘roughness’ of the front, introducing non‐linearities into the spread and making invasion trajectories harder to predict (Azimzade, 2022). Over time, these dispersal‐enhancing traits can evolve rapidly – sometimes within just a few generations – provided they are not constrained by high gene flow at the invasion front swamping local adaptation (Beer *et al*., 2024). This evolutionary acceleration can drive spread dynamics beyond what would be expected from environmental drivers alone (Ochocki & Miller, 2017). High dispersal propensity is commonly favoured under such conditions, resulting in populations at the invasion front being disproportionately composed of individuals that move further, more frequently, and with greater directionality (Alford *et al*., 2009; Brown, Phillips & Shine, 2014). Conversely, traits such as increased competitive ability and resource efficiency could emerge in more densely populated core areas (Messager & Olden, 2019). This process of spatial sorting differs from classical natural selection in which traits that facilitate range expansion are promoted, irrespective of how the underlying genes affect the survival of an organism or its reproductive success (Shine *et al*., 2011). In plants, the patchiness of suitable habitat itself can have an important influence on how selection acts on dispersal and competitive ability at the invasion front. As gaps between patches increase in size, non‐native species may rapidly evolve greater maximum dispersal distances, driven by selection on dispersal‐related traits such as seed morphology and mass, display of dispersal structures, and plant height for more efficient seed release, especially in wind‐dispersed species (Monty & Mahy, 2010; Thomson *et al*., 2011; Williams *et al*., 2016).

A comprehensive understanding of the spatial progression of the invasion front also requires the integration of genetic perspectives (Szűcs *et al*., 2017; Kołodziejczyk *et al*., 2025). The genetic diversity in a non‐native population may even exceed that of the native range due to many individuals introduced following multiple introduction events originating from various source populations, leading to admixture (Roman & Darling, 2007). Populations at the core of an invaded area often experience distinct genetic dynamics compared to those at the periphery. Populations at the expanding front are typically subject to various interrelated processes, such as serial founder effects, population bottlenecks, and genetic drift. These processes can reduce genetic diversity, which can limit adaptive potential (Day, 2015). In some cases, the amplification of these dispersal traits can come at a cost, ultimately affecting individual fitness (Kelehear & Shine, 2020) and accompanied by genetic costs (i.e. reduced genetic diversity, decline of fitness‐related traits, etc.; Peischl *et al*., 2015). The low population density and high growth rate at invasion fronts during serial founder effects may intensify genetic drift, allowing new but also standing mutations to increase in frequency, regardless of whether they are neutral, benign, or deleterious, creating a so‐called ‘expansion load’ (Klopfstein, Currat & Excoffier, 2006; Peischl *et al*., 2013). Lowered adaptive potential at the front can further be influenced by the non‐random accumulation of alleles associated with increased dispersal, even if these alleles come with the cost of reduced fitness (Hoffmann, Sgrò & Kristensen, 2017). Gene flow from core to front populations, however, adds further complexity as it may replenish genetic diversity and introduce adaptive alleles that bolster performance at the invasion front (Berthouly‐Salazar *et al*., 2013), thereby mitigating the negative effects of founder events and expansion load. However, excessive gene flow can also swamp locally adapted genotypes, impeding local adaptation and slowing the rate of spread (Beer *et al*., 2024). The low genetic diversity at the front can also rapidly increase through hybridisation or introgression events, potentially expanding the adaptive potential (Hessenauer *et al*., 2020). Thus, the genetic architecture of invasion fronts plays a dual role, as it not only shapes the evolutionary trajectory of non‐native populations but also influences the pace and success of range expansion (Szűcs *et al*., 2017).

Taken together, the invasion front should not be viewed merely as the geographic boundary of expansion, but as a biologically active and sometimes behaviourally distinctive zone, shaped by the complex interplay of dispersal strategies, eco‐evolutionary dynamics, population structure, and landscape context (Dominguez Almela *et al*., 2022; Beer *et al*., 2024). A narrow focus on dispersal alone may obscure these interactions and lead to an underestimation of the forces driving invasion momentum and the pace of non‐native spread (Grayson & Johnson, 2018).

### (3) Modulators of non‐native species spread

Species spread is profoundly shaped by a diverse and interrelated set of modulators, spanning abiotic, biotic, and anthropogenic factors. These modulators can interact with the intrinsic traits of the spreading species to constrain, amplify, or diversify the dynamics of range expansion, leading to spatial, temporal, and species‐specific variation in non‐native spread (Bradley *et al*., 2024). Abiotic modulators of spread can influence the permeability of the environment to dispersal, and include landscape connectivity (Caplat *et al*., 2016), habitat heterogeneity (Melbourne *et al*., 2007), environmental gradients (Bradley *et al*., 2024), disturbance regimes, and the suitability of neighbouring areas (Davis, Grime & Thompson, 2000). Competition, predation, mutualisms, pathogens, and density dependence are among the plethora of biotic modulators affecting species spread, which can in turn influence species demographics and establishment success along a spreading population. Anthropogenic modulators, such as habitat fragmentation, construction of linear structures (e.g. canals), or barrier removal (Leuven *et al*., 2009; Dolan *et al*., 2025; Andrade‐Restrepo, Levin & Rodríguez‐Iturbe, 2019*b*; Chapman *et al*., 2020) can further catalyse range shifts. For instance, connecting previously isolated river catchments in Europe, comprising 30 main inland canals and over 100 branches, has effectively removed barriers to species spread (Soto *et al*., 2023*b*). Collectively or individually, these modulating factors can lead to transitions between wave‐like and discontinuous spread patterns (Fraser *et al*., 2015). In addition to evolutionary and ecological drivers, endogenous demographic processes such as over‐compensatory growth, Allee effects, and density‐dependent dispersal can also cause spatiotemporal fluctuations in the progression of invasions (Sullivan *et al*., 2017; Ochocki & Miller, 2017), producing pulses, slowdowns, or chaotic progression, even in the absence of external environmental changes. Moreover, invasion science also considers how anthropogenic environmental change and other global stressors (e.g. marine litter; Haram *et al*., 2021) reshape species distributions.

#### (a) Environmental change and land‐use alterations

Spatiotemporal shifts in climatic conditions, such as increasing temperatures, changes in precipitation regimes, glacier retreats, and altered seasonality, are globally reshaping the distribution of organisms (Ficetola, Thuiller & Miaud, 2007; Bradley *et al*., 2024; Vergés *et al*., 2014). For populations to persist under these changing environmental conditions, they must either adapt (e.g. physiological adaptation and phenological alterations), shift their geographic ranges to track ecological niches, or both, resulting in range shifts. Notably, shifting climate envelopes can enable non‐native species to expand into areas that were previously unsuitable, blurring the line between ‘natural’ spread and environmental change‐induced range shifts (Bellard *et al*., 2013, 2013; Early *et al*., 2016; Essl *et al*., 2019; Mitchell & Dominguez Almela, 2025). Traits facilitating rapid spread, broader climatic tolerances, and ongoing human‐mediated introductions collectively enable non‐native species to spread more rapidly and either persist or expand more effectively in response to global environmental change, conferring a competitive advantage over native species (Bradley *et al*., 2024). This interaction of spread with environmental change could be even more important in poikilothermic animals, mainly in temperate and polar ecosystems, since the predicted increase in temperature will increase metabolism, hatching success, and activity (with possible payoffs in higher spread rates) of these animals unless the temperature exceeds the maximum thermal tolerance threshold. In addition, climate change may shift the temporal window for possible spread in these poikilothermic animals (Walther *et al*., 2002), and, importantly, enable more generations per year or successful overwintering (Veselý, Buřič & Kouba, 2015; Lewkiewicz *et al*., 2022). However, the opposite may happen in many species that mainly spread during their larval phase, since higher temperatures will reduce their larval duration and consequently their larval dispersal. In freshwater systems, climate change may increase salinity, favouring non‐native species that are more halotolerant than natives (e.g. Carbonell *et al*., 2017). In addition, environmental fluctuations also affect establishment and thus spread (e.g. Cuddington & Hastings, 2016). Concurrently, land‐use changes such as urban expansion, deforestation, and agricultural intensification are fragmenting natural habitats while creating novel niches. These combined pressures can enable non‐native species to colonise areas that were previously uninhabitable due to climatic or ecological constraints (Bellard *et al*., 2013*b*; Berdugo *et al*., 2020). Conversely, emerging stressors, including droughts or land degradation, may impose new limitations, potentially contracting current ranges or creating dispersal bottlenecks (Diez *et al*., 2012). Extreme flooding or fire events can facilitate the spread of non‐native animals or plants into habitats where they were absent previously (Everts *et al*., 2025). When environmental changes intersect with human‐mediated dispersal vectors, they may generate non‐linear and discontinuous spread patterns, complicating prediction and management (Early *et al*., 2016; Lembrechts *et al*., 2016).

#### (b) Species‐specific traits and biotic interactions

The dynamics of range fronts and spread potential are strongly influenced by species‐specific traits, such as reproductive strategy, dispersal ability, environmental tolerance, and behavioural plasticity. For example, plant invasiveness is often associated with maximum plant height for enhanced competition for light, greater seed output, and high specific leaf area for faster photosynthetic activity and resource assimilation (Martín‐Forés *et al*., 2023). Plants that disperse successfully in general have wider niche breadths and broader areas of occupancy in their native regions, with their range size appearing correlated to both greater height and larger specific leaf area (Sporbert *et al*., 2021). Moreover, Paudel *et al*. (2025) found that plants that expand faster in their non‐native range were also those expanding their native range. Besides, widespread annual plants can display greater biomass, shifts in phenological cycles, and increased plasticity in the invaded range for rapid adaptation and enhanced invasiveness (Martín‐Forés *et al*., 2017, 2018, 2018). Such species‐specific traits can interact with demographic stochasticity and biotic interactions, including competition, predation, parasitism and diseases, and facilitation processes (Thuiller *et al*., 2010). In a predator–prey context, for instance, both predators and prey can generate heterogeneity for one another (i.e. endogenous biotic heterogeneity). The rate of spread of predator and prey species is thus tightly linked to the local density of the other: whereas predator spread is modulated by prey availability, prey spread is influenced by predator pressure (Melbourne *et al*., 2007). In a competition context, invasion speed decreases with increasing competition intensity, with negligible range expansions when both competitors are equally strong (Hastings *et al*., 2005). Such ecological constraints to spread align with the ‘biotic resistance theory’, which posits that high biodiversity and resulting biotic interactions with local communities can limit, but rarely completely prevent, the establishment, local population growth, and subsequent spread of advancing non‐native populations (Levine, Adler & Yelenik, 2004). However, empirical support for the biotic resistance hypothesis is outweighed by studies that question it (Jeschke *et al*., 2012; Enders *et al*., 2020) demonstrating that its validity is context dependent (Beaury *et al*., 2020) and influenced by spatial scale, with negative relationships often observed at small scales but positive ones at larger scales (Byers & Noonburg, 2003).

Related to these inter‐specific interactions is the process of secondary invasions, where the influence of a primary invader facilitates the establishment and spread of a subsequent secondary invader (O'Loughlin & Green, 2017). Conversely, previously established non‐native species can also contribute to biotic resistance, especially when they compete with or even exclude subsequent non‐native species or limit their success at establishing successfully. Importantly, range boundaries often do not represent hard ecological limits but are transitional zones of low density, where populations are especially sensitive to environmental variability and stochastic disturbances (e.g. Allee effects). Adaptive trait evolution also plays a key role. For example, evolutionary changes in dispersal and life‐history traits can accelerate (i.e. adaptation) or decelerate (i.e. maladaptation) spread, especially at invasion fronts, where selection pressures differ markedly from those in established populations (Phillips *et al*., 2008*a*). Such eco‐evolutionary feedbacks may either reinforce invasiveness and further entrench non‐native species within novel ecosystems or erode adaptive potential and thereby possibly non‐native spread.

#### (c) Environmental heterogeneity and resource distribution

Spatiotemporal environmental heterogeneity, particularly in the form of patchiness in habitat quality or resource availability, is a fundamental determinant of spatiotemporal invasion trajectories (Melbourne *et al*., 2007). When resources are unevenly distributed or autocorrelated across landscapes or over time, the speed and success of range expansion can be significantly affected. For instance, higher resource clumping has been shown to slow down invasion fronts, especially for species that exhibit resource‐seeking behaviour or whose population dynamics are governed by strong demographic stochasticity (Giometto, Altermatt & Rinaldo, 2016). Asynchronised dynamics among local populations can inflate regional population growth and facilitate spread through portfolio effects: a stabilising effect when different populations respond differently to environmental changes (Hui, Fox & Gurevitch, 2017). Moreover, spatial variability in microclimate, hydrology, habitat suitability, or topography can interact with species' niche requirements, producing invasion pathways that are irregular and locally constrained. Natural dispersal of non‐native species tends to advance most rapidly in the slipstream of the path of least resistance (i.e. suitable environmental conditions), but this momentum can decelerate once this geographical space is colonised, compelling populations to expand into suboptimal habitats (Urban *et al*., 2008; Everts *et al*., 2025). As species approach the spatial limits of their potential niche, suboptimal environments can constrain the further expansion of an invasion and demarcate a stable range boundary, particularly when the niche is conceptualised as static rather than dynamic (Holt, Barfield & Gomulkiewicz, 2005; Pagel & Schurr, 2011). Theoretical predictions that overlook environmental influences across multiple spatial scales may therefore yield inaccurate estimates of non‐native spread (Urban *et al*., 2008).

#### (d) Dispersal dynamics and clustering thresholds

The pattern of spread is modulated by dispersal‐related traits and the structure of the environment. A key concept here is the ‘clustering threshold’: when dispersal costs are high (or long‐distance dispersal mechanisms are not available), intraspecific competition occurs over short distances, or steep environmental gradients exist, spread tends to occur in discrete clusters or pulses rather than as a continuous wave (Andrade‐Restrepo *et al*., 2019*a*). Below this threshold, spread tends to occur in discrete clusters or pulses. Above this threshold, populations exhibit smoother, wave‐like expansion. These thresholds are determined by the interaction of intrinsic species attributes (e.g. energy allocation to movement or growth) and extrinsic landscape features (e.g. habitat permeability), highlighting the need for multiscale approaches in predicting spread dynamics (With, 2002).

#### (e) Anthropogenic factors and dispersal corridors

Human activities play a dual role in either directly or indirectly shaping invasion trajectories: they can result in species introductions into new regions and subsequently influence how those species spread. Linear infrastructures, such as roads, railways, water transfer infrastructure, and canals, can act as dispersal corridors for terrestrial and aquatic invaders, providing connectivity between discrete habitat patches, often enabling long‐distance jump dispersal events, and potentially facilitating rapid change expansion (Hulme, 2009; Lembrechts *et al*., 2016; Anson & Pickering 2013; Waine, Robertson & Pattison, 2024; Phillips *et al*., 2024). Transport networks, such as hiking trails, migrating ungulates, or shipping routes, further ensure a constant influx of non‐native species (Manzano & Malo, 2006; Koyama, Egawa & Akasaka, 2025; Andrés *et al*., 2023). Beyond transport and infrastructure, intentional movements linked to trade in ornamental fishes, aquarium releases, exotic pets, and ornamental plants or landscaping represent additional pathways; repeated introductions *via* these sectors can strongly accelerate the spread of non‐native species along human‐created corridors (Hulme *et al*., 2018). However, the presence of barriers (e.g. dams but also other physical structures) can compromise the continued spread of many freshwater organisms (Dolan *et al*., 2025). It should also be noted that dams can simultaneously function as hot spots for introductions and stepping stones once a barrier has been overcome (Johnson, Olden & Vander Zanden, 2008). Furthermore, these corridors often intersect environmental gradients or disturbance regimes, intensifying invasion risks (e.g. the network of inland canals in Europe; Nunes *et al*., 2015). Environmental change may amplify these interactions by altering corridor suitability or changing the timing of dispersal events (Hufbauer *et al*., 2012). Urbanisation can further enhance non‐native species spread by altering landscape connectivity, modifying physical properties of the environment, and interacting with cultural, socioeconomic, biogeographic, and climatic factors (reviewed in Potgieter *et al*., 2024), while accelerating evolutionary processes that may affect non‐native spread (Briski *et al*., 2025). Integrated management strategies must therefore account for both landscape configuration and species behaviour, addressing the socio‐ecological context in which invasions unfold.

#### (f) Debris and novel dispersal substrates

**Fig. 6:**
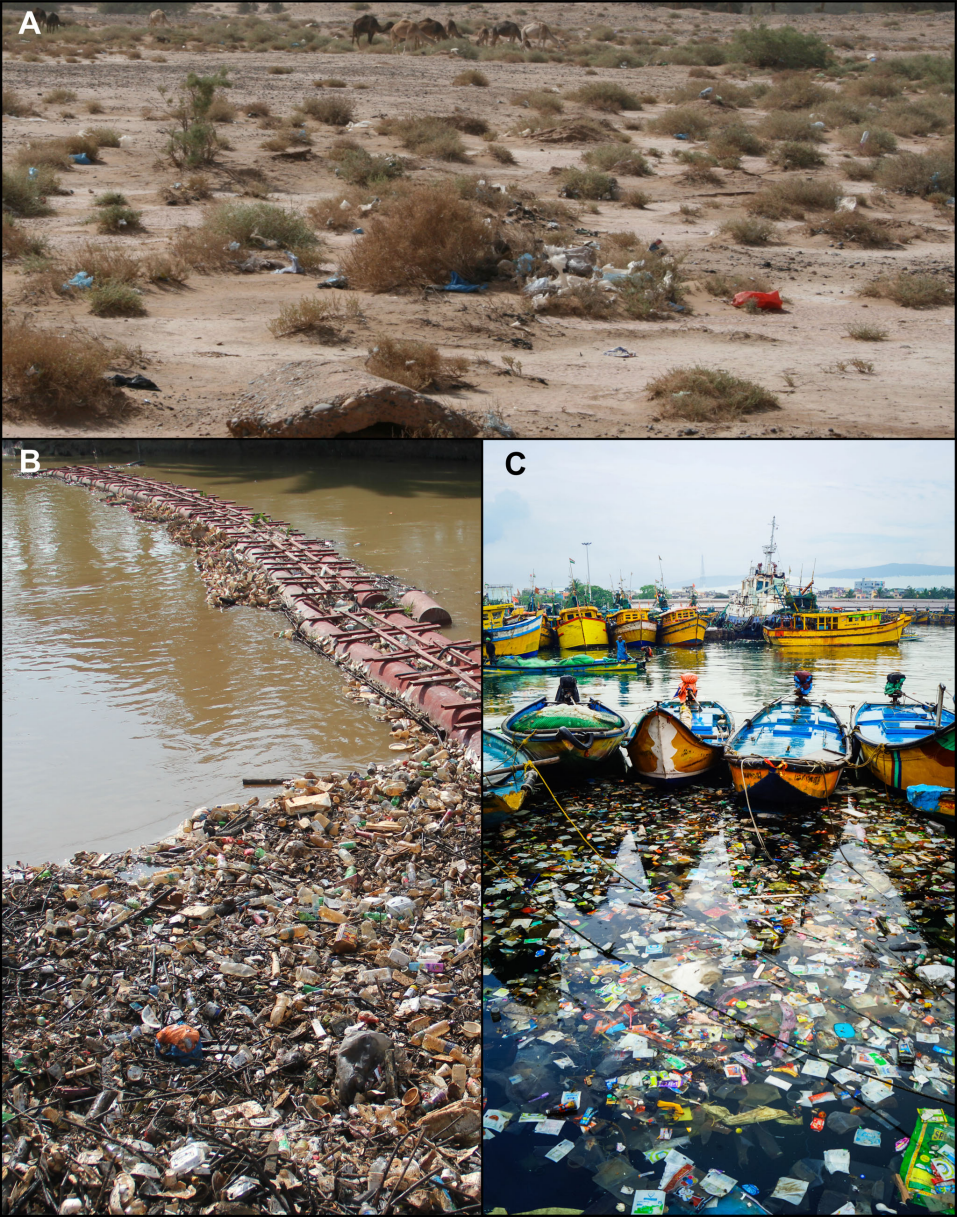
In parts of the Sahara Desert (Morocco), the accumulation of plastic can be so high that it is plausible to assume that it may aid in the dispersal of non‐native insects or other organisms (A; photograph by Ronaldo Sousa). In South‐East Asian rivers (B; photograph of a river in Malaysia by Ronaldo Sousa) and oceans (C; photograph by Gude Pavan on Pixabay), plastic debris is increasingly recognised as a persistent vector facilitating the long‐distance transport of non‐native species across aquatic ecosystems.

The proliferation of waste in marine environments has introduced an unprecedented vector for long‐distance dispersal of non‐native species. Unlike natural flotsam, synthetic debris – especially plastics – can persist for decades, providing durable, buoyant substrates that facilitate the transport of sessile and rafting organisms across vast oceanic distances (Kiessling, Gutow & Thiel, 2015). This phenomenon, sometimes referred to as ‘rafting invasions’, extends beyond passive dispersal: it enables the establishment of novel mid‐ocean and coastal communities composed of both native and non‐native taxa, with implications for biogeography, ecosystem function, and biosecurity. The global scale and persistence of plastics effectively create a synthetic seascape that bypasses traditional dispersal limitations, particularly for species with limited pelagic larval phases. In combination with ocean currents and climatic events (e.g. storms, tsunamis), these substrates can deliver viable propagules to remote or previously uncolonised regions, complicating surveillance and early detection efforts (Carlton & Fowler, 2018). In an extreme case, adult barnacles were found attached to plastic leg rings of migratory gulls, suggesting that long‐distance bird migration (and their interaction with plastic) can inadvertently transport marine invertebrates across continents (Tøttrup *et al*., 2010). As such, plastic debris represents both a symptom of anthropogenic environmental degradation and a novel modulator of biological invasions in primarily marine systems. Although similar results may occur in freshwater and terrestrial ecosystems (Fig. 6), research in these systems is still in its infancy. However, the movement of potted ornamental plants and soil has facilitated the introduction and spread of land planarians (Geoplanidae), free‐living carnivorous flatworms that act as apex predators in soils (Sluys, 2016; Fourcade, Winsor & Justine, 2022). Movement of contaminated soil, packaging, garden waste machinery, and boots also facilitates long‐distance spread of many micro‐organisms and plants (Valls *et al*., 2014; Helsen *et al*., 2021; Ormsby, 2022) but also of amphibians by floating meadows (Fonte *et al*., 2021).

#### (g) Pathogens can modulate host spread

Throughout the invasion process, a symbiotic complement (e.g. a microbiome/symbiome/pathobiome) is an unavoidable component in the spread of a non‐native host (Bojko *et al*., 2023*b*). All organisms house microbial symbioses, some of which impose pathogenic relationships and reduce the health and lifespan of the non‐native host (Bojko *et al*., 2023*a*). This therefore affects the capacity of non‐native species to persist in and impact the new environment (Carneiro *et al*., 2025). At the transportation stage of an invasion, many pathogenic species tend to be lost through ‘enemy release’ (Miura & Torchin, 2023): these are either left in the native range of the host as the propagule moves to a new location, or the pathogen may cause infected individuals to die before they arrive, influencing propagule size upon arrival. Co‐non‐native pathogens (‘non‐native parasites’) may be lost at the establishment stage of an invasion, possibly due to incompatible climate, ecology, density dependence, and/or other transmission factors (Dunn *et al*., 2012), benefiting host health. If a pathogen persists in the established population, it can benefit the host by acting as a biological control agent (Vilcinskas, 2015), reducing competition to facilitate spread, or it can alternatively reduce the host's health and population size, limiting the capacity for spread (Romeo *et al*., 2025).

#### (h) Mutualist microbiomes

The biotic interactions underpinning invasions extend beyond visible species assemblages to include co‐invading microbial mutualists, with significant implications for community structure and ecosystem functioning (Romeo *et al*., 2025). For example, in the case of plants, native and non‐native legumes are associated with distinct rhizobial lineages enabling, among others, nitrogen fixation. Non‐native *Acacia* spp. rely on compatible rhizobial symbionts, which often co‐invade or are recruited from local pools, restructuring the existing microbial communities in the process (Le Roux & Wandrag, 2023). As non‐native *Acacia* spp. can dominate the community, the rhizobial microbiome associated with native plants shifts in composition (Le Roux, Mavengere & Ellis, 2016), harbouring less diverse and more compositionally homogeneous communities, suggesting community filtering or competitive exclusion (Kamutando *et al*., 2019). Compositional shifts can extend beyond the rhizobial community; for example, invasion by *Acacia dealbata* reshaped bacterial and fungal communities, ultimately resulting in lower richness and functional diversity not only belowground but also for understory plant communities (Lazzaro *et al*., 2014), whereas buffel grass (*Cenchrus ciliaris*) overcomes nutrient limitation in arid environments by modifying the soil microbiome to include more nitrifiers, arbuscular mycorrhizal fungi, ectomycorrhizal fungi, and methanotrophs in invaded compared to uninvaded soil (Aslani *et al*., 2019; Gornish *et al*., 2020). Microbiome‐mediated feedbacks can also trigger functional shifts, such as soil acidification and nitrification or reduced soil moisture (Vietorisz *et al*., 2025) or even facilitate further establishment of non‐native plant species through shared microbiomes, resulting in a mutualism‐mediated co‐invasion cascade that is difficult to detect with traditional biodiversity surveys.

### (4) Environmental differences

#### (a) Spread in terrestrial ecosystems

Terrestrial spread is shaped by a complex interplay of abiotic and biotic features acting at the landscape scale (With, 2002). These features include topography, connectivity, habitat heterogeneity, lithological substrates, soil properties, vegetation architecture, and anthropogenic landscape modifications, which together interact to filter, redirect, or amplify dispersal across space and time, resulting in complex, scale‐dependent spread dynamics. As a result, the terrestrial matrix is fragmented and anisotropic, with dispersal pathways governed by the permeability across mosaics of different land uses and properties (Murphy & Lovett‐Doust, 2004), and their interaction with spatial filters (Vasudev *et al*., 2015). These filters include, among others, topographical constraints (Shepard *et al*., 2013), slope, elevation (Stage & Salas, 2007; Seipel *et al*., 2016; Di Musciano *et al*., 2022), gravitational transport, hydrological flow, and the behavioural decisions of mobile species (Gruber & Peckham, 2009; Bouchet *et al*., 2015). Valleys can act as dispersal corridors funnelling mobile animals or wind‐blown seeds, while ridgelines or steep escarpments deflect or stall spread and change exposure to solar radiation and prevailing winds (Stage & Salas, 2007; Singh, 2018; Ginal *et al*., 2021). Lithology and geomorphic substrate influence species spread through cascading effects on soil development, drainage patterns, vegetation structure, and animal movement (Ott, 2020). Rocky or compact substrates can limit root penetration, plant colonisation, burrowing, and animal locomotion (Ducey *et al*., 1993; Unger & Kaspar, 1994), whereas friable or weathered substrates generally promote deeper, more heterogeneous soils that support richer vegetation, enhance propagule retention, and attract diverse dispersers like frugivores and herbivores (Muller‐Landau & Hardesty, 2005). Substrate also influences animal movement—affecting gait, refuge access, and predator exposure—thereby shaping dispersal corridor suitability. Soil texture, moisture, and temperature impact plant establishment and ground‐dwelling or fossorial fauna behaviour. For example, compact soils can hinder seed germination and insect movement, while loose soils aid fossorial activity and nesting (Ducey *et al*., 1993). Moisture and surface temperature further influence movement timing in ectotherms, especially juveniles and egg‐laying stages.

Superimposed on the abiotic matrix, vegetation structure and configuration influence spatial heterogeneity, permeability, and biotic interactions affecting organism movement, refuge, and spread. Vegetation complexity shapes microhabitat continuity, modulates interspecific interactions (e.g. competition, predation, disease transmission), and determines energetic travel costs (Melbourne *et al*., 2007; Boon *et al*., 2023; Vimercati *et al*., 2024). Dense canopies can hinder large‐bodied dispersers, reduce visibility for predators, limit wind dispersal, and restrict light‐demanding species, but support arboreal and shade‐tolerant taxa *via* habitat continuity. Open or patchy vegetation facilitates rapid seed and vertebrate movement (Gabay, Perevolotsky & Shachak, 2012) but can limit the spread of shade‐ or humidity‐dependent species (e.g. amphibians, molluscs). Many non‐native species are *r*‐strategists, for which disturbance‐driven fragmentation (e.g. fire, grazing) reduces competition and opens structural pathways (Tilman, 2004; Gabay *et al*., 2012). Sessile species are more dependent on abiotic/biotic vectors, making spread sensitive to environmental flows and connectivity across fragmented habitats, aided by anthropogenic corridors like roads (Tewksbury *et al*., 2002; Uroy, Ernoult & Mony, 2019). Mobile species respond based on behaviour, range, and edge tolerance; fragmentation may limit *K*‐strategists but benefit generalists and synanthropes (Bowler & Benton, 2005; Baguette & Van Dyck, 2007). Spread is further shaped by species‐specific interactions (Bonte, Keith & Fronhofer, 2024) and behavioural traits like territoriality and foraging range (Börger, Dalziel & Fryxell, 2008; Tao, Börger & Hastings, 2016; Toscano *et al*., 2016). Temporal factors (e.g. seasonal pulses, phenology, disturbances) create dynamic windows of spread (Measey, 2016), highlighting that terrestrial spread arises from a complex interplay of physical, biological, and anthropogenic forces.

#### (b) Spread in freshwater ecosystems

Non‐native species spread in freshwater ecosystems is affected by the structural and ecological characteristics of these habitats and occurs following a fundamentally different set of challenges, conditions, and constraints, compared to other environments (Strayer, 2010). Freshwater systems are characterised by a variable mosaic of highly connected but also isolated habitats. Rivers and streams form intrinsically (directionally and hierarchically) connected dendritic networks, whose complexity can alter invasion dynamics (Dominguez Almela *et al*., 2022), presenting natural corridors that can facilitate both downstream and upstream movement (Goldberg *et al*., 2010; Everts *et al*., 2025). As human settlements have historically clustered around fresh water (Postel & Carpenter, 1997), the alteration and exploitation of freshwater ecosystems (e.g. aquaculture, shipping, recreational fisheries, the aquarium trade, and the construction of canals and reservoirs) resulted in their widespread degradation (Padilla & Williams, 2004; Gherardi, 2007). Hydrological dynamics thus act as dispersal conduits, especially in arid zones (Chesson *et al*., 2004; Fraaije *et al*., 2015), while mesic systems offer continuous water but stronger biotic resistance. Upstream movement is often locally impeded by physical barriers, flow dynamics, and usually higher energetic costs (van der Walt *et al*., 2016). By contrast, lakes and ponds are more isolated insular habitats where introduction events and subsequent colonisation rely on external natural vectors, such as waterfowl (Green & Wilkinson, 2024), or anthropogenic vectors such as aquaculturists, anglers, boats, and aquarists, particularly for obligately aquatic species (Drake & Lodge, 2004; Gozlan *et al*., 2010; Oficialdegui *et al*., 2025).

These contrasts within freshwater ecosystems result in different non‐native species spread dynamics, with rivers promoting linear, often rapid, expansions and lakes requiring stochastic jump dispersal events, and in larger lake ecosystems spread occurring either along the shore or diffusely throughout the open water and deeper zones (Havel, Lee & Vander Zanden, 2005). Indeed, spread in freshwater ecosystems is also shaped by vertical and lateral gradients: non‐native species may expand from littoral to pelagic zones, from shallow to deep areas, or from tributaries into main channels following environmental gradients such as light, temperature, oxygen, or substrate composition (Karatayev, Burlakova & Padilla, 2002). These gradients interact with life‐history traits, dispersal mechanisms, and habitat preferences to create spatially complex and ecologically heterogeneous spread. For riverine systems, predictive approaches have revealed how river network complexity can interact with species' dispersal traits to influence spread rates, demonstrating that higher habitat connectivity and introduction location strongly determine invasion success and front progression (Dominguez Almela *et al*., 2022). Crucially, much of this activity occurs below the surface, making it difficult to detect with conventional monitoring and delaying responses to incipient invasions (Ficetola *et al*., 2007; Keller, Frang & Lodge, 2008). Moreover, terrestrial dispersal adds another layer of complexity, as some non‐native species like crayfish and amphibians are capable of moving between isolated water bodies across terrestrial barriers following specific cues (such as overpopulation, limited resources, cannibalism, temperature; Edeline *et al*., 2025), but also facilitated during periods of elevated air humidity, rain or fog, especially at night (Herrmann, Schnabler & Martens, 2018; Measey, 2016). In temporary aquatic systems common in semi‐arid environments, non‐native invertebrates and fish can undergo repeated recolonisation and extinction dynamics (Zylstra *et al*., 2019), whilst invertebrates with an egg bank can persist during dry periods (Coccia *et al*., 2016). Particularly interesting, but mostly ignored and unquantified, may be the impact of extreme events, such as floods, on the spread of freshwater non‐native species such as fish into formerly endorheic lakes (Lipták *et al*., 2016; Maceda‐Veiga, Mac Nally & De Sostoa, 2017), or that of amphibian tadpoles into geographically more isolated pondscapes (Everts *et al*., 2025). If these events become more frequent and intense, they could significantly influence the spread rate, especially in the downstream direction (Diez *et al*., 2012).

#### (c) Spread in marine ecosystems

The mechanisms that drive non‐native species spread in the ocean differ substantially from those that operate on land. Unaided oceanic dispersal is the dominant pathway for secondary spread, often surpassing the importance of primary introductions (Katsanevakis *et al*., 2020). Most marine organisms have a dispersive life stage, such as planktonic larvae, eggs, spores, or other propagules, that can drift on ocean currents and colonise distant habitats (Cowen & Sponaugle, 2009). Passive larval dispersal along prevailing currents explains the rapid expansion of several marine invaders (e.g. Schilling *et al*., 2024). However, larval behaviour, including active swimming and vertical migration, can significantly influence dispersal patterns and population connectivity, challenging the traditional view of larvae as passive drifters (Cowen, Paris & Srinivasan, 2006). The lack of empirical data on larval behaviour, particularly vertical swimming capabilities, significantly hampers accurate modelling of non‐native species dispersal in new marine environments, as such behaviours can profoundly influence larval trajectories and connectivity patterns (Gary *et al*., 2020). Regions with high connectivity, whether due to prevailing currents, stepping‐stone habitats like archipelagos, or man‐made structures, such as seawalls, piers, and offshore installations, are particularly vulnerable to rapid spread (Adams *et al*., 2014; Bishop *et al*., 2017). Mobile species such as fishes or crabs can also actively swim or crawl into new areas, but in most cases, such active movement is augmented by the passive dispersal of early life stages *via* currents.

Beyond natural dispersal, a significant proportion of secondary spread is driven by human activities, as the amount of trade through shipping continues to increase (Hulme, 2009, 2021). Regional ship traffic can transfer non‐native species rapidly across long distances, creating a mosaic of newly colonised regions (Costello *et al*., 2022). Next to ballast water (David & Gollasch, 2015), hulls of recreational vessels are particularly significant for non‐native species spread, often cited as the largest unregulated human vector for the spread of marine non‐native species (Clarke Murray, Pakhomov & Therriault, 2011; Ashton *et al*., 2022). Marinas serve as stepping stones for marine non‐native species by providing suitable habitats and facilitating their regional spread by connecting distant locations through recreational boating networks (Ulman *et al*., 2019). Fishing gear, such as trawl nets and trammel nets, can serve as a vector for the secondary spread of non‐native species. In the case of the non‐native alga *Caulerpa taxifolia*, such equipment facilitated the transport of algal propagules across regions, thereby contributing to the establishment of new invasion fronts (Relini, Relini & Torchia, 2000). Sessile non‐native species may exploit natural vectors for long‐distance movement, such as rafting on floating seaweed, driftwood, or pumice (Gracia, Rangel‐Buitrago & Flórez, 2018). Plastic litter is an emerging human‐mediated vector, which may increase the potential for successful oceanic spread by increasing the duration and distance of dispersal beyond what would be possible otherwise (Haram *et al*., 2021; Rech *et al*., 2025). Until recently, most natural rafts have tended to degrade or sink within months to a few years. By contrast, marine plastic debris can persist for decades, altering historical dispersal limitations by enabling long‐distance and long‐term transport for a wide range of non‐native species (Carlton *et al*., 2017; Haram *et al*., 2021).

Physical barriers to spread in the ocean are fewer than on land, but they are not insignificant. Among the most critical constraints to dispersal are abiotic factors, especially depth, temperature, and salinity (Jaspers, Møller & Kiørboe, 2011; Dimitriadis *et al*., 2020; Castellanos‐Galindo *et al*., 2025), which, in the absence of adaptation or spatial sorting, often define the invasion front. Climate change is increasingly acting both as a catalyst and a modifier of marine invasions. Rising ocean temperatures drive the ‘tropicalisation’ of temperate regions, relaxing thermal barriers that once restricted the spread of warm‐water species (Wesselmann *et al*., 2024). Moreover, marine heatwaves can function as pulse events that may accelerate spread. These heat waves impose physiological stress or mass mortalities on native species (Garrabou *et al*., 2022) while creating favourable conditions for heat‐tolerant non‐natives, which may then outcompete, displace, or replace resident taxa (Atkinson *et al*., 2020; Spyksma, Miller & Shears, 2024). Nevertheless, due to the difficulty in defining native ranges in marine ecosystems, where native distributions are often largely assumed, the dispersal of marine organisms remains understudied compared to terrestrial and freshwater species and most invasions remain cryptic (Carlton & Schwindt, 2024), highlighting the need for future research.

#### (d) Aerial spread

In birds, as in other organisms capable of flight, vagrancy is commonplace (Dufour *et al*., 2024), allowing individuals to reach beyond their native range and, in some cases, to colonise new areas on their own under the influence of human‐mediated environmental changes (Steeves *et al*., 2010). In other instances, birds have been deliberately introduced to new continents (e.g. as ornamental species) and subsequently spread naturally, partly through migratory flights. Some species have caused conservation issues in areas far removed from their original points of introduction (e.g. ruddy ducks, *Oxyura jamaicensis*, spreading from the UK to Spain; Muñoz‐Fuentes, Green & Negro, 2013). In some non‐native birds, spread through flight coincides with spread *via* the pet trade, making it difficult to disentangle these mechanisms (e.g. parakeets in Europe; Strubbe & Matthysen, 2009). Numerous aquatic insects also possess the ability to fly, which allows them to disperse much more rapidly than by water alone (Ortego *et al*., 2021). Some non‐native spider species can disperse longer distances by ‘ballooning’, the process of releasing silk threads that catch the wind and lift them away (Malumbres‐Olarte *et al*., 2014). Moreover, waterbirds are a known factor for the dispersal of aquatic non‐native species (Reynolds, Miranda & Cumming, 2015).

### (5) The Allee effect

The Allee effect refers to a phenomenon in population ecology where individual fitness or population growth rates decline at low population densities, constituting a form of positive density dependence (Kramer *et al*., 2009; Stephens, Sutherland & Freckleton, 1999). Allee effects can emerge from various mechanisms, including mate limitation (i.e. difficulty in locating reproductive partners in sparse populations, or males pairing with another, more‐abundant species when conspecific females are rare), reduced cooperative behaviours such as group foraging or predator defence, and failures in social facilitation (Kuussaari *et al*., 1998; Grayson & Johnson, 2018). In the context of biological invasions, these effects may also arise when only a small number of individuals from a non‐native species are introduced on a single or few occasions into a new area (i.e. low propagule pressure), thereby increasing the species' susceptibility to stochastic processes (Drake & Lodge, 2006). Indeed, when propagule pressure is low, random fluctuations in birth rate, death rate, and sex ratio (i.e. demographic stochasticity), as well as extreme weather events, floods, and fires (i.e. environmental stochasticity), become major determinants of establishment success (Simberloff, 2009).

Superimposed on these stochastic influences is the genetic composition of the introduced individuals (Sakai *et al*., 2001). Non‐native populations originating from low propagule pressures typically are genetically impoverished (Roman & Darling 2007). In the short term, limited genetic diversity within small founding populations can give rise to inbreeding depression, thereby reducing individual fitness and diminishing the likelihood of successful establishment, particularly in animals and self‐incompatible plants (Allendorf & Lundquist, 2003; Fauvergue *et al*., 2012). Over time, genetic variation may further erode through genetic drift and consequently constrain the adaptive potential of established populations in response to environmental change. This could stem from environmental mismatches between native and introduced ranges, from expansion into ecologically distinct regions within the invaded range (Beer *et al*., 2024), isolation after an initial colonisation (Belouard *et al*., 2019), or from emerging pressures such as environmental change, increased inter‐ and intraspecific competition as the population grows, or anthropogenic management interventions (Abdelkrim, Pascal & Samadi, 2007). While genetic diversity is key for populations to prosper (Crawford & Whitney, 2010), it is not an exclusive determinant for introduced populations to succeed (Roman & Darling, 2007). For instance, the influence of reduced genetic diversity on establishment success is less substantial for asexually reproducing organisms. Examples include the parthenogenetic marbled crayfish (*Procambarus virginalis*), the Japanese knotweed (*Reynoutria japonica*), and numerous aquatic plants with the ability to spread rapidly from vegetative fragments (Barrat‐Segretain, Bornette & Hering‐Vilas‐Bôas, 1998; Maiakovska *et al*., 2021; Wang *et al*., 2025).

For sexually reproducing organisms, the negative effects of genetic impoverishment can be overcome when these are associated with traits that benefit invasiveness, such as the loss of intraspecific colony competition in Argentine ants (*Linepithema humile*) (Tsutsui *et al*., 2000; Tsutsui, Suarez & Grosberg, 2003). More commonly, outbreeding of rare deleterious alleles can easily be purged by selection in small, inbred populations (Kristensen & Sørensen, 2005), while phenotypic plasticity – where one genotype can express different phenotypes – can confound the relationship between genetic diversity and establishment success (Geng *et al*., 2016). While there are many other mechanisms that can prevent a lowered genetic diversity and the subsequent detrimental effects – such as hybridisation, self‐fertilisation, high reproductive output, and polyploidy – inbreeding × environment interactions can effectively allow genetically impoverished populations to overcome the effects of genetic depletion and inbreeding depression (Schrieber & Lachmuth, 2016). Whether or not non‐native populations originating from low propagule pressures and consequent reduced genetic diversity will affect their spread is thus context and taxon dependent, and far from completely understood.

Populations experiencing strong Allee effects exhibit a critical density threshold, known as Allee threshold, below which population growth becomes negative, potentially leading to extirpation even in otherwise favourable environments (Boukal & Berec, 2002; Berec, Angulo & Courchamp, 2007). This has important implications for invasion dynamics, particularly at the invasion front, where densities tend to be low (Bøhn *et al*., 2004; Brandner *et al*., 2013; Raffard *et al*., 2022; Alves *et al*., 2025) and populations vulnerable to demographic collapse. Under such conditions, Allee effects may suppress establishment success following long‐distance dispersal events or slow down the rate of spread by reducing numerical growth at the advancing edge (Lewis, 1997; Travis & Dytham, 2002). Notably, Allee effects can mitigate the otherwise accelerating expansion seen in ‘fat‐tailed’ dispersal kernels – distributions that allow for rare, long‐distance dispersal movements – by limiting the success of these colonisation events (Clark, Lewis & Horvath, 2001). Furthermore, when Allee effects interact with over‐compensatory population growth, density‐dependent dispersal, or heterogeneous landscapes, they can produce complex, non‐linear invasion dynamics marked by pulsed or even chaotic spread (Gregory *et al*., 2010; Sullivan *et al*., 2017). Beyond invasions, Allee effects have broad ecological and management relevance. They can affect metapopulation persistence, determine species' range boundaries, influence the success of reintroduction programs, and even shape the spread of infectious diseases (Fernandez, Hance & Deneubourg 2012; Taylor & Hastings, 2005). Incorporating Allee dynamics into models of species spread is therefore essential for accurate forecasting and the development of effective control strategies.

## III. BENEFITS OF UNDERSTANDING NON‐NATIVE SPREAD

Understanding how non‐native species spread is critical for management strategies aimed at preventing new introductions, containing an invasion or mitigating their ecological, economic, and social impacts. Recognising the mechanisms and modulators of spread (see above) enables more accurate predictions, better informed policy, and deeper ecological and evolutionary insights into the nature of biological invasions.

### (1) From prediction to policy and management

Forecasting the potential spread of non‐native species is essential for early detection, risk identification, assessment, and resource prioritisation. However, the practical value of such forecasts hinges on their predictive accuracy (e.g. see the failure to predict the spread of the lionfish (*Pterois miles*) in the Mediterranean Sea; Johnston & Purkis, 2014). Conventional approaches have typically relied on diffusion‐based or correlative models, which often rest on some simplifying assumptions. Recently, more sophisticated models have emerged that incorporate species‐specific traits, dispersal kernels, density dependence, Allee effects, and eco‐evolutionary dynamics. These more advanced models have demonstrated significantly improved performance in capturing the complexity of spreading non‐native species (Sullivan *et al*., 2017; Andrade‐Restrepo *et al*., 2019, 2019) but are more complex and data demanding. Predictive models must also address questions of how, where, and how fast invasions will occur, including scenarios of continuous *versus* pulsed expansion or, wave‐front *versus* leap‐frog (stratified) dynamics (Lewis, 1997; Travis & Dytham, 2002).

Recent advancements have led to the development of some tools designed primarily for risk identification and screening rather than comprehensive risk assessment. These include the Aquatic Species Invasiveness Screening Kit (AS‐ISK; Vilizzi *et al*., 2021), which is applicable to aquatic organisms; the Terrestrial Animal Species Invasiveness Screening Kit (TAS‐ISK; Vilizzi *et al*., 2022); and the Terrestrial Plant Species Invasiveness Screening Kit (TPS‐ISK; Vilizzi *et al*., 2024), which are tailored for terrestrial animals and plants, respectively. These tools serve as valuable first steps in identifying potentially non‐native species and help prioritise candidates for further, more detailed risk assessment. In this context, risk assessment frameworks have become more sophisticated. Among these, the recently introduced Dispersal‐Origin‐Status‐Impact (DOSI) scheme (Soto *et al*., 2024; Błońska *et al*., 2024; Tarkan *et al*., 2024*a*; Haubrock *et al*., 2025) allows assessing biological invasions at the population level, rather than generalising across species. DOSI incorporates four key dimensions – dispersal mechanisms, origin, current status, and impact – allowing for more refined assessments tailored to ecosystem‐specific and regional contexts. It is particularly useful in distinguishing between expanding and static populations, autonomous *versus* human‐assisted spread, and context‐specific ecological impacts. By accounting for dynamic and nuanced invasion trajectories, DOSI improves the objectivity and utility of prioritisation schemes. Other established assessment tools like Environmental Impact Classification for Alien Taxa (EICAT) (Hawkins *et al*., 2015) and the European Non‐native Species in Aquaculture Risk Analysis Scheme (ENSARS) (Tarkan *et al*., 2020) focus more broadly on species‐level invasiveness and environmental risk. Integrating these schemes with DOSI could offer a more holistic risk framework, combining cross‐ecosystem generalisability with population‐specific detail. Recent advances in spatially explicit statistical models (e.g. Gaussian Process Gradient Models; Goldstein *et al*., 2019) or spatially explicit individual‐based models (IBMs) such as those implemented in *RangeShifter* (Dominguez Almela *et al*., 2022) now allow for localised estimates of spread, capturing not only the presence of invasion risk but also its trajectory and momentum. These models can be fitted to empirical data using inverse modelling approaches such as approximate Bayesian computation, which infer (otherwise difficult to obtain) population and dispersal parameters (Dominguez Almela *et al*., 2020). This integration of process‐based simulation with empirical calibration enables more reliable identification of invasion hotspots, dispersal corridors and conditions driving expansion rates. Further advancement in predictive approaches also enables the simulation of eco‐evolutionary dynamics over temporally changing landscapes, incorporating dispersal evolution, population genetics, and management interventions in a fully spatially explicit and individual‐based framework (Bocedi *et al*., 2021).

Management actions are only as effective as the ecological understanding on which they are based. Real‐world invasions are complex, non‐linear, and influenced by stochasticity and feedback. Non‐native plants may disperse much further than predicted by classical dispersal syndromes (González‐Varo *et al*., 2024). Allee effects, for instance, may mask population expansion at early stages, creating a false sense of containment that might be suddenly overcome by a threshold‐crossing event (Berec *et al*., 2007; Spear *et al*., 2021). Similarly, spatial sorting and evolution of dispersal‐enhancing traits can rapidly alter invasion dynamics, reducing the efficacy of static buffer zones or fixed monitoring boundaries (Alford *et al*., 2009; Phillips *et al*., 2008*b*). Frameworks like DOSI provide a clear management advantage. By ranking non‐native populations based on their spread dynamics and local impacts, DOSI allows for tailored interventions and prioritisation at the scale of specific ecosystems or habitats. Unlike species‐level tools, it can flag sleeper populations (*sensu* Spear *et al*., 2021) that may not yet show large‐scale impacts but are poised for rapid expansion when triggered by an environmental factor. This population‐level resolution is especially valuable in freshwater systems, where complex networks of natural and artificial dispersal pathways (e.g. canals, stocking, aquaculture escapees) complicate management.

Model‐based evidence supports the prioritisation of early interventions at invasion fronts, where local abundance is still low. Simulations have shown that such strategies are more effective than targeting core populations, especially for fast‐dispersing species in dendritic river systems (Dominguez Almela *et al*., 2021). Using process‐based models such as IBMs demonstrates the use of explicitly testing management scenarios to identify optimal interventions and trade‐offs in controlling non‐native species (Samson *et al*., 2017; Dominguez Almela *et al*., 2021). Furthermore, incorporating socio‐economic factors, as proposed in refinements to DOSI, is essential. Cost–benefit analysis of non‐native species control, such as trade‐offs between ecological restoration and stakeholder interests (e.g. recreational fishing), must guide policy. This integrative approach helps balance ecological integrity with economic feasibility and social acceptance (Bradshaw *et al*., 2024).

### (2) Ecological and evolutionary insights

Beyond management, the spread of non‐native species offers a unique lens through which to explore fundamental ecological and evolutionary processes underlying range expansions of any kind. Invasion fronts function as natural experiments where dispersal evolution, trait filtering, and species–environment interactions play out in real time (Travis & Dytham, 2002; Alford *et al*., 2009). Traits such as boldness, directional persistence, and low site fidelity, but also self‐compatibility and genome size, are often selected at the front, offering direct evidence of spatial sorting and selection gradients (Myles‐Gonzalez *et al*., 2015; Pannell *et al*., 2015; Pizzatto, Child & Shine, 2017*b*; Cang *et al*., 2024). Moreover, spread patterns can reveal critical ecological thresholds, for example, in community resistance mutualistic networks, or habitat saturation. They can also reveal unexpected dispersal mechanisms, such as the spread of dry‐fruited plants by migratory birds (Martín‐Vélez *et al*., 2021). Range dynamics also help identify areas of ecological release *versus* environmental constraint, providing insight into niche conservatism *versus* niche shift debates (Wiens & Graham, 2005; Broennimann *et al*., 2007). Understanding these dynamics helps contextualise not only how non‐native species move but also why some succeed, and others fail, underpinning broader theories of colonisation, adaptation, and ecological resilience (MacDougall & Turkington, 2005; Sax *et al*., 2007; Davidson, Jennions & Nicotra, 2011). Finally, spread processes offer valuable lessons for global change biology. As environmental (including climate) change shifts habitat suitability, alters disturbance regimes, or drives glacial retreats, species with high dispersal and adaptive capacity are likely to respond first and fastest, making the study of spread a leading indicator for future ecological transformation (Walther *et al*., 2009; Bellard *et al*., 2013*b*; Capinha *et al*., 2015; Ficetola *et al*., 2024).

## IV. METRICS AND MODELS FOR CAPTURING SPREAD

### (1) Measuring spread: observations, rates, and rare events

Quantifying the spread of non‐native species is a central challenge in invasion science. Spread is inherently spatially and temporally heterogeneous, shaped by a wide range of ecological, evolutionary, and stochastic processes. As a result, no single metric can fully capture the complexity of an invasion's trajectory (Pyšek & Hulme, 2005). Commonly used measures, such as maximum range size or area of occupancy (Weber, 1998), or more recently range size, local abundance, and habitat breadth (Fristoe *et al*., 2021), offer useful snapshots, but they often obscure underlying dynamics such as non‐linear rates, long‐distance dispersal, or multiple introduction points (Mineur *et al*., 2010). Kernel density estimates and convex/alpha hull methods have been widely used in mapping occupied ranges to mitigate the sparsity and spatial heterogeneity of occurrence records (Fleming & Calabrese, 2017; Burgman & Fox, 2003). Increasingly, species distribution models incorporating environmental covariates are also being used to project invaded ranges (e.g. Formoso‐Freire *et al*., 2023). The cumulative (or spontaneous) spread rate is typically represented by a spread curve of the distance of the first occurrence record (or the first breeding record) to the presumed introduction location (e.g. Orledge, Smith & Reynolds, 2010; Hui *et al*., 2012), the square root of the area occupied (e.g. Silva, Reino & Borralho, 2002; Mitikka *et al*., 2008), or the range radius (e.g. Choi & Park, 2012; Siegert *et al*., 2014), along the time axis (typically in years). Slope changes along the spread curve, or its form, estimated from linear, non‐linear, additive, quantile regressions, can be used for hypothesis testing on whether the spread is linear, exponential, sigmoidal, biphasic, in acceleration or lag phase, exhibits a boom‐and‐bust pattern, or whether the spread dynamics are concordant or discordant among different invasion events, introduction points, and range radiuses (Hui & Richardson, 2017; Osunkoya *et al*., 2021).

One of the most critical insights from past research is the disproportionate influence of rare long‐distance dispersal events, captured in fat‐tailed or leptokurtic dispersal kernels, on overall spread dynamics (Lewis, 1997; Neubert & Parker, 2004; Petrovskii & Morozov, 2009). Even when infrequent and often human mediation is underlying, these events can seed distant colonies, effectively circumventing local resistance and accelerating the pace of an invasion (Everts *et al*., 2025). This makes traditional diffusion‐based spread models inadequate for many real‐world scenarios. Moreover, invasions may follow elastic patterns, with bursts of expansion followed by periods of stasis or reorganisation. This occurs when dispersal dynamics interact with adaptation lags and environmental gradients, resulting in highly context‐dependent and temporally variable invasion speeds (Andrade‐Restrepo *et al*., 2019*a*), even for the same species (Haubrock *et al*., 2024). In such cases, the spread is not just complex, it defies simplification. Importantly, invasion trajectories can be strongly shaped by the demographic structure of the population itself. Modelling stage‐structured populations (where individuals differ in traits such as dispersal probability, survival, or fecundity) can reveal key processes that otherwise are hidden in unstructured approaches (Cockrell & Sorte, 2013; Lewkiewicz *et al*., 2022). Neglecting this heterogeneity risks misidentifying the life stages most responsible for range shifts, leading to less‐effective management strategies and underestimation of spread potential.

To capture this complexity, novel data sources are increasingly being integrated into invasion monitoring and modelling frameworks. One of the most impactful developments has been the rise of citizen science, which has significantly improved our understanding of the spatiotemporal distributional patterns, abundance, species–environment associations, and movements of non‐native species through massive data generation (Pocock *et al*., 2024; Fajgenblat *et al*., 2025). Platforms such as *eBird* and *iNaturalist* now aggregate millions of geo‐referenced observations contributed by volunteers, providing high‐resolution data that reveal dynamic patterns of range expansion, seasonal movements, and local establishment (Dyer, Redding & Blackburn, 2017; Lourenço *et al*., 2024). These data sets have proved particularly valuable for identifying early invasion events, multiple locations from which spread independently initiates, long‐distance dispersal, and transient populations that may otherwise escape formal surveillance (De Bona *et al*., 2023; Belouard *et al*., 2025; Everts *et al*., 2025). Moreover, citizen science broadens geographic and temporal coverage, helping to fill critical data gaps in invasion science (Price‐Jones *et al*., 2022). In addition, the use of local ecological knowledge may also help in the assessment of the spread of non‐native species (Azzurro *et al*., 2019; Marchessaux *et al*., 2023). When used in conjunction with structured surveys, bias correction, and modelling, these data enable more precise tracking of spread in space and time (Fajgenblat *et al*., 2025).

Complementing these observational advances, molecular techniques – particularly Next Generation Sequencing – have introduced new capabilities for reconstructing the genetic and demographic history of biological invasions (Estoup *et al*., 2010; Kołodziejczyk *et al*., 2025). By comparing genetic variation and rates of gene flow among populations in the introduced range, Next Generation Sequencing approaches can estimate rates and directions of spread, identify source populations, and detect multiple or secondary introductions (Ortego *et al*., 2021; Everts *et al*., 2025). This is particularly useful for cryptic or under‐recorded taxa, where visual records are sparse or species identification is unreliable (Dufresnes *et al*., 2024). Genomic data can also be used to infer the timing of introductions and historical population dynamics, offering insight into the tempo and mode of invasion (Estoup *et al*., 2010; Estoup & Guillemaud, 2010). Together with citizen science data, these genetic tools form a powerful complementary set of methods that enrich our capacity to measure, model, and ultimately manage the spread of non‐native species (Sherpa *et al*., 2020; Everts *et al*., 2025; Davinack, 2025).

However, empirical data remain a central bottleneck in improving spread models. Disparities in recorded rare dispersal events, cryptic invasions, missing information, and multiple introduction points often obscure the true trajectory of range expansion. Although citizen science, local ecological knowledge, remote sensing, genomic tools, high‐resolution occurrence data (e.g. from platforms like *eBird*; Dyer *et al*., 2017; Lourenço *et al*., 2024), culturomics and internet ecology (Jaric *et al*., 2021) and genomic reconstructions of spread history can supplement sparse field data and capture undocumented long‐distance dispersal or reintroduction events (Pocock *et al*., 2024; Everts *et al*., 2025), these data currently remain insufficient to predict future spread of non‐native species.

### (2) Modelling spread: from theory to prediction

Modelling the spread of non‐native species is a foundational component of invasion science. Widely used in modelling the spread of non‐native species are dynamic system models and implementation schemes. Commonly used models in this context are ordinary, partial, and stochastic differential equations, stochastic processes, and more generically integro‐difference or integro‐differential equations. Popular implementation schemes include grid‐based lattices such as cellular automata, network and gravity models, and agent/individual‐based modelling, as well as correlated random walks or Levy flights or hybrid models that integrate mechanistic spread processes with environmental suitability. Early models were grounded in the reaction–diffusion partial differential equation, first proposed by Skellam (1951), which described spread as a function of two core parameters: the intrinsic rate of increase (r, more generically; the *per‐capita* growth rate when rare, also known as the invasion growth rate) and a diffusion coefficient (d), assuming random movement in a homogeneous environment. Under these assumptions, the invasion front expands at a constant velocity ($C=2\sqrt{\mathrm{rd}}$). While analytically tractable, this model fails to capture the irregular, often patchy dynamics of invasions, particularly those influenced by long‐distance jump dispersal, environmental heterogeneity, or directional spread mechanisms. An emerging challenge in invasion spread modelling is capturing non‐linear and temporally variable dynamics. Fluctuations in spread rates can arise from internal feedbacks, such as Allee effects, density‐dependent dispersal, or over‐compensatory population growth, which may produce pulsed, patchy, or even chaotic spread (Sullivan *et al*., 2017; Ochocki & Miller, 2017). These dynamics underscore that spread is not always continuous or wave‐like, but often irregular and contingent on interactions between invader traits and environmental context (Milanović *et al*., 2020). To address these limitations, more sophisticated modelling approaches have been developed. Integro‐difference equations, and their stage‐structured extensions, incorporate life‐history traits, environmental stochasticity, and dispersal variation, offering more flexible and biologically realistic projections of spread (Neubert & Parker, 2004; Bogdan *et al*., 2021).

A key refinement was the incorporation of long‐distance dispersal into modelling frameworks, which introduced fat‐tailed or leptokurtic dispersal kernels to account for rare but influential jump events (Kot, Lewis & van den Driessche, 1996). These models recognise that infrequent long‐range dispersal can lead to the establishment of distant satellite populations, which may grow and merge with the main front, accelerating the overall invasion speed (Shigesada & Kawasaki, 2016). This process of stratified diffusion highlights how traditional diffusion models may underestimate invasion velocity or fail to predict sudden range expansions (Clark *et al*., 2001). To overcome this, spatially explicit statistical tools such as Gaussian process gradient modelling have emerged as powerful alternatives (Goldstein *et al*., 2019). These models use time‐of‐arrival data to estimate the local speed and direction of spread, allowing researchers to identify not only sites of long‐range jumps but also how environmental features (e.g. host availability, habitat structure) modulate invasion dynamics.

However, despite their importance, long‐distance dispersal events are notoriously difficult to detect or quantify empirically (Hastings *et al*., 2005; but see Belouard *et al*., 2025; Everts *et al*., 2025) and often are indistinguishable from human‐mediated secondary introductions. Modern approaches thus shifted toward spatially explicit and biologically more realistic models. Integrodifference equation models, for example, allow the incorporation of variable dispersal kernels, stage structure, and habitat heterogeneity (Neubert & Parker, 2004), simulating spread under conditions that include environmental stochasticity, biotic interactions, or demographic constraints. Similarly, individual‐based models incorporate eco‐evolutionary feedback, dispersal behaviour, and demographic processes at the population level (Henry *et al*., 2014; Fraser *et al*., 2015; Dominguez Almela *et al*., 2022). Individual‐based models are particularly valuable in this context, as they explicitly incorporate local density dependence in both demographic and dispersal processes, enabling the simulation of emergent complex spread patterns over time (Bocedi *et al*., 2021). For instance, recent applications of IBMs have shown that young individuals (i.e. not yet mature) may play a disproportionately large role in driving early expansion fronts (Dominguez Almela *et al*., 2020). Meanwhile, Gaussian process gradient models use observed time‐of‐arrival data to estimate spatial gradients of spread and detect landscape features that shape dispersal (Goldstein *et al*., 2019).

Beyond classical diffusion‐based and individual‐based models, a growing suite of integrative tools is now used to predict the spread of non‐native species, reflecting the increasing complexity of ecological data and landscape structure (Table 2). One of the most widely adopted approaches remains species distribution modelling, which commonly uses occurrence data and environmental predictors to estimate habitat suitability across space, but also models that explicitly consider occurrence records that are delineated both spatially and temporally (Elith, 2017; Dobson *et al*., 2023). While traditional species distribution models [e.g. MaxEnt, BIOMOD (Thuiller *et al*., 2009; Phillips *et al*., 2017)] assume that suitable habitat implies potential occupancy, they often overpredict spread by ignoring dispersal constraints, temporal lags, and biotic interactions. To address this, hybrid species distribution models now incorporate dispersal kernels, landscape resistance, or are coupled with process‐based models to simulate range expansion over time, accounting not just for where a species can survive, but how and when it may arrive (Barber‐O'Malley *et al*., 2022; Gardner *et al*., 2024). In parallel, network‐based models conceptualise landscapes as connected nodes, such as watersheds, ports, or transit corridors, where species move through structured, often human‐mediated pathways (Ashander *et al*., 2022). These models excel at identifying spread bottlenecks or invasion hubs that are often missed by continuous‐space frameworks.

**Table 2:** A non‐exhaustive list of models commonly used to study the spread of non‐native species.

Tool/model	Function/case	Reference
Reaction–diffusion models	Simulate spread *via* local diffusion and growth; assume homogeneous environments	Skellam (1951, 1991)
Integrodifference equation models	Incorporate life‐history traits and variable dispersal across space	Neubert & Parker (2004)
Stratified diffusion	Models combining short‐range diffusion and long‐distance jumps	Kot *et al*. (1996); Clark *et al*. (2001)
Individual‐based models	Simulate individual demography and dispersal behaviours, spatial feedbacks and eco‐evolutionary dynamics	Bocedi *et al*. (2021); Dominguez Almela *et al*. (2022)
Gaussian process gradient models	Use time‐of‐arrival data to estimate local spread speed and direction	Goldstein *et al*. (2019)
Species distribution models	Predict suitable habitat based on environmental variables; correlative	Elith & Leathwick (2009)
Hybrid species distribution models (with dispersal)	Combine SDMs with dispersal kernels or resistance layers for spread prediction	Engler & Guisan (2009)
Network‐based models	Simulate spread through connected nodes (e.g. rivers, ports, road networks)	Vicente *et al*. (2016)
Cellular automata	Rule‐based spread across spatial grids; ideal for simple landscape processes	Parks *et al*. (2005)
Gravity/radiation models	Estimate spread likelihood based on ‘mass’ and distance; often human‐mediated vectors	Leung *et al*. (2006); Song *et al*. (2024); Spadon *et al*. (2024)
Machine learning	Identify non‐linear relationships in large data sets; predict invasion risk	Carter *et al*. (2021); Elias (2023)
Bayesian hierarchical models	Integrate multiple data types with uncertainty estimation	Wikle (2003); Hooten & Wikle (2008)
State‐space models	Separate process noise from observation error; useful with time‐series or sparse data	Damgaard *et al*. (2011); Nishimoto *et al*. (2021)
Epidemiological models (e.g. SIR)	Track spread of agents through space or networks; adaptable to invasion systems	Gilligan & van den Bosch (2008); Ferrari *et al*. (2014)

Machine learning techniques, including random forests, neural networks, and ensemble algorithms, offer another layer of insight by detecting non‐linear associations among species presence, environmental gradients, and anthropogenic drivers (Araújo & New, 2007; Zhang, Yang & Wang, 2024). Though largely correlative (Kearney, Wintle & Porter, 2010; Horemans *et al*., 2024), such models can flag high‐risk invasion areas with high predictive power when trained on large data sets. Meanwhile, Bayesian hierarchical and state‐space models provide a flexible statistical structure to integrate multiple data types, i.e. occurrence, abundance, genetics, or citizen science, while explicitly accounting for uncertainty in both biological processes and observation (Froese, Pearse & Hamilton, 2019; Malchow *et al*., 2023). Parameters of mechanistic and generative models can also be estimated and identified, for instance, using Bayesian inference and deep learning, to reconstruct past and forecast future range expansion under invasion scenarios (Botella *et al*., 2018, 2022; Formoso‐Freire *et al*., 2023).

## V. WAY FORWARD

### (1) Innovative potential

The study of non‐native species spread is being transformed by technological innovation, cross‐disciplinary integration, and the pressing need for real‐time, spatially explicit forecasting tools. As statistical and, in particular, predictive models grow in complexity (Soto *et al*., 2025), there is increasing convergence with frameworks used in epidemiology, particularly those developed in response to emerging pathogens like COVID‐19 (Nuñez *et al*., 2020). Approaches such as SIR (Susceptible–Infectious–Recovered) models, agent‐based simulations, and network‐based spread models, implemented in platforms like *GLEAMviz* (Broeck *et al*., 2011), *EpiModel* (Jenness, Goodreau & Morris, 2018), or *Covasim* (Kerr *et al*., 2021), may offer valuable conceptual and computational blueprints for future tools used to predict the spread of non‐native species. Because these tools incorporate high‐resolution mobility data, contact structures, and intervention scenarios, they could be adapted to ecological analogues such as habitat connectivity, human‐mediated dispersal routes (i.e. contact networks; Perry, Moloney & Etherington, 2017), or management interventions (Ashander *et al*., 2022). Moreover, gamified platforms like *Plague Inc*. illustrate the conceptual power of trait‐based modelling and intervention testing in dynamic environments that could be adapted to predict the spread of non‐native species (Jacques, 2015; Mitchell & Hamilton, 2018). Concomitantly, the emergence of novel sensing and surveillance technologies such as drones, satellite imagery, camera traps, biosensors, robotics, and environmental DNA (eDNA) sampling, often supported by artificial intelligence, is revolutionising data acquisition (Allard *et al*., 2023; Meira *et al*., 2024; Everts *et al*., 2024; Katsanevakis *et al*., 2024). Computer vision, supported by machine learning, now allows for real‐time species identification and spread mapping from high‐volume visual data sets (Høye *et al*., 2021; Dyrmann *et al*., 2024). Integration of these technologies with citizen science platforms like *iNaturalist* or *eBird* can further broaden the geographic and taxonomic scope of non‐native monitoring (Callaghan *et al*., 2022; Lourenço *et al*., 2024), thus helping to identify long‐distance dispersal events.

Finally, artificial intelligence (AI), particularly large‐scale Foundation Models and geospatial machine learning, is emerging as a potentially transformative approach for forecasting non‐native species spread (Reynolds *et al*., 2025). These models, trained on multimodal data sets that can include ecological, climatic, geospatial, and trade data, can integrate diverse inputs to predict invasion risk across taxa and landscapes (Guo *et al*., 2025). Recent models such as Prithvi‐EO‐2.0 developed by IBM and NASA, are trained on over 4.2 million global time‐series samples from the Harmonised Landsat‐Sentinel archive and use temporal and spatial embeddings to power geospatial understanding across tasks like land‐use classification and vegetation monitoring (Szwarcman *et al*., 2024). Similarly, SkySense, a multimodal geospatial foundation model, integrates optical and Synthetic Aperture Radar (SAR) time‐series data, improving performance on spatiotemporal tasks relevant to landscape‐level ecological monitoring (Guo *et al*., 2024). These models have already demonstrated the capacity to identify subtle patterns in Earth Observation data, offering new tools for mapping species range shifts and habitat suitability in real time. For example, foundation models pretrained on remote sensing data are beginning to outperform traditional machine learning models in predicting ecological outcomes, especially in data‐sparse or dynamically changing environments (Strong *et al*., 2025). While challenges remain in terms of interpretability, training efficiency, and bias correction (Morera, 2024), Foundation Models have already demonstrated the capacity to identify patterns, deriving embeddings from Earth Observation data, and showing strong potential for mapping range shifts and habitat suitability in real time (Guo *et al*., 2024; Schmude *et al*., 2024). Coupling these models with reinforcement learning frameworks may further enable optimisation of management strategies under constraints, for example minimising invader abundance or cost. Dietterich, Taleghan & Crowley (2013) demonstrated the application of reinforcement learning in managing non‐native plant species by developing policies that balance control efforts with resource limitations.

### (2) From prediction to action

While there is debate over whether the spread of invasions is inherently unpredictable, implying that all existing models may have a fundamental limit to their accuracy (Beckage, Gross & Kauffman, 2011), AI‐driven approaches could introduce novel and potentially unforeseen predictive capabilities (Silvestro *et al*., 2022). For example, digital twins (i.e. a digital representation of a physical object or process that can be continuously updated; de Konning *et al*., 2023) applied to ecological processes could hold particular promise for assessing invasive species spread. By creating virtual replicas of ecosystems, these models can integrate real‐time data, simulate scenarios, and predict outcomes, potentially supporting management strategies (Khan *et al*., 2024). Nevertheless, model performance is ultimately constrained by the quality and resolution of underlying ecological data, particularly regarding dispersal and recruitment dynamics, and by the inherent stochasticity of ecological processes. Beyond forecasting, therefore, consideration of non‐native species spread naturally leads to questions of management and control. Indeed, a growing body of literature highlights that determining where and how to intervene and manage spreading non‐native species is not only an ecological question but also increasingly one of economic and logistical nature. Optimisation of management strategies is thus highly context dependent as it is shaped by factors such as landscape structure, invasion geometry, management budget, and the spatial distribution of damage. For effective invasion control and containment, strategies must be both spatially targeted and forward‐looking, with efforts focused on minimising exposed invasion edges and anticipating the directions of greatest potential spread and damage (Epanchin‐Niell & Wilen, 2012). Such approaches can substantially reduce long‐term containment costs, particularly when the geometry of the landscape and the spatial dynamics of spread are taken into account. However, implementation is often complicated by the realities of complex, heterogeneous land‐use systems. In so‐called ‘management mosaics’ where land and water are subdivided among diverse stakeholders, successful management depends on coordinated decision‐making (Epanchin‐Niell *et al*., 2010). Without aligned incentives, individual inaction can create persistent sources of reinvasion that undermine broader regional efforts. Moreover, even once a non‐native species is established, determining the economically optimal management strategy remains a substantial challenge. Optimal management requires integrating knowledge of spread dynamics, control costs, and potential damage into decision‐making frameworks that account for uncertainty and spatial complexity (Epanchin‐Niell & Hastings, 2010). Decisions such as whether to target satellite populations or core invasion zones therefore ultimately depend not on general rules, but on context‐specific features such as landscape configuration, invasion extent, and the spatial distribution of costs and benefits.

## VI. CONCLUSIONS

(1) Understanding and managing the spread of non‐native species is central to addressing the broader threat presented by biological invasions. While introductions initiate invasions, it is the capacity of non‐native species to spread across space, time, and ecosystem boundaries that modulates their ecological, economic, and societal impacts.
(2) The inherent dynamics of non‐native species spread are shaped by a complex interplay of species traits, environmental conditions, and human activities. Due to the intertwined functioning of dispersal mechanisms and factors underlying recruitment success (i.e. environmental changes, land‐use alterations, and the availability of anthropogenic vectors), invasion trajectories are multifactorial, non‐linear, and context dependent.
(3) Current management frameworks often underestimate or oversimplify spread, focusing too narrowly on presence or impact. A shift towards dynamic, population‐level assessments is needed for more realistic and actionable evaluations of invasion risks, emphasising dispersal origin and mechanisms, directionality, and spread momentum.
(4) Improved forecasting of spread requires more investment in on‐ground standardised long‐term and large‐scale monitoring data, as well as better integration of data, tools, and technologies. This includes leveraging spatially explicit models, genomic insights, citizen science, environmental DNA, culturomics and internet ecology, remote sensing, and AI to detect and anticipate spread in real time, especially in understudied regions and taxonomic groups.
(5) Coordinated management responses must reflect the variability of spread dynamics and support adaptable, context‐specific strategies. Strengthening cross‐sector collaboration and investing in consistent monitoring and data‐sharing frameworks may improve our ability to detect, predict, and manage the spread of non‐native species across regions and ecosystems.

## Data Availability

Data sharing not applicable to this article as no datasets were generated or analysed during the current study.
